# The Global Spread of Microplastics: Contamination in Mussels, Clams, and Crustaceans from World Markets

**DOI:** 10.3390/foods13233793

**Published:** 2024-11-26

**Authors:** Tamara Mutić, Jelena Mutić, Miloš Ilić, Vesna Jovanović, Jelena Aćimović, Boban Andjelković, Dragana Stanić-Vucinić, Maria Krishna de Guzman, Mirjana Andjelkovic, Mirjana Turkalj, Tanja Cirkovic Velickovic

**Affiliations:** 1 Center of Excellence for Molecular Food Sciences, Faculty of Chemistry, University of Belgrade, Studentski trg 12-16, 11000 Belgrade, Serbiajmutic@chem.bg.ac.rs (J.M.); ilicm@chem.bg.ac.rs (M.I.); vjovanovic@chem.bg.ac.rs (V.J.);; 2 Center for Food Biotechnology and Microbiology, Ghent University Global Campus, Incheon 21985, Republic of Korea; mariakrishna.deguzman@ugent.be; 3 Risk and Health Impact Assessment, Sciensano, Juliette Wytsmanstreet 14, 1050 Brussels, Belgium; 4 Srebrnjak Children’s Hospital, HR-10000 Zagreb, Croatia; mturkalj@bolnica-srebrnjak.hr; 5 Faculty of Medicine, Josip Juraj Strossmayer University of Osijek, HR-31000 Osijek, Croatia; 6 School of Medicine, Catholic University of Croatia, HR-10000 Zagreb, Croatia; 7 Serbian Academy of Sciences and Arts, Knez Mihajlova 35, 11000 Belgrade, Serbia

**Keywords:** MPs, microFTIR, digestion, Crustacea shellfish, bivalves, commercial seafood

## Abstract

Analysis of microplastic (MP) occurrence in commercially relevant species is a prerequisite for food risk assessment. Using a standardized methodology, we aimed to investigate MP contamination in point-of-sale clams, mussels, and Crustacea shellfish collected from various markets (Belgium, Croatia, Serbia, and South Korea). An improved digestion protocol yielded ≥ 99.8% digestion efficiency for all species analyzed. In a total of 190 samples analyzed individually by microFTIR, MPs were identified in 43.68% of the samples with less than 1 MP/individual average (0–4 MP/individual, 0–1.35 MPs/g tissue). Significant differences between species were observed when considering samples contaminated with MPs, with Crustacea shellfish having the lowest MPs/g of edible tissue. Polystyrene, polypropylene, and polyethylene were dominant MPs found in clams and mussels, while polyamide, polyethylene terephthalate, and polyvinyl chloride were the most abundant in Crustacea shellfish. Our data do not support the bioaccumulation or biomagnification of MPs with the size of the animal in the shellfish group. MP contamination is more strongly associated with the type of shellfish than with the geographical origin of the market.

## 1. Introduction

Microplastics (MPs) are persistent and widespread environmental contaminants that have so far been detected in soil, drinking water, and food products for human consumption [1,2]. MPs are defined as plastic fragments of a size less than 5 mm, originating from a variety of sources [3]. They are derived from synthetic polymers that are either initially manufactured in microscopic sizes (primary MPs), such as exfoliating agents in cosmetic products, or generated via the breakdown of large plastic fragments (secondary MPs) due to chemical, physical, and biological processes [1,4]. Globally, 92.4% of the plastics in the oceans are MPs [5], making it the most prevalent form of marine plastic pollution. Eriksen et al. [5] estimated that at least 5.25 trillion plastic particles weighing 268,940 tons are currently floating at sea. Their omnipresence and durability have raised safety concerns [6]. While the full extent of health implications remains to be conclusively determined, the omnipresence of MPs in various food products, drinking water, and air underscores the urgency of addressing this issue, particularly regarding the effects of MPs in the gastrointestinal tract, such as inhibition of food assimilation and/or decreased nutritional value of food [7]. It has been shown that MPs of various polymer types can bind to and alter the structure and functional properties of the main egg white protein [8]. MPs binding to pepsin direct its enzymatic action, and the small size polystyrene MPs reduced pepsin activity [9].

The presence of MPs in the food chain and transmission to humans has been documented in many studies [10,11]. Ingestion is nowadays assumed to be the main route of human exposure to MPs [12], highlighting the need for more profound studies on MPs occurrence in raw and processed food products to assess the real magnitude of human exposure through ingestion of foodborne MP, coupled with the release of MPs from plastic packaging. Exposure estimates are difficult, as the data available in the literature are hard to compare and are insufficient for a reliable assessment of MP ingestion [13,14,15,16]. The assessment of the food safety risk related to MP contamination is, consequently, impeded [2].

Seafood is recognized as a significant vector for MP transfer to humans, highlighting the direct implications for public health and the urgent need for comprehensive investigations into the extent of MP contamination across various types of seafood [15,17]. Recent studies employing different methodologies to isolate, identify, and quantify MPs in marine organisms, from shellfish and crustaceans to fish and seaweeds, revealed a picture of MPs contamination that varies significantly across species, geographical locations, and aquatic environments [18]. For food risk assessment, analysis of occurrence and complete characterization of morphological and chemical characteristics of MPs in commercially relevant species and their edible parts (flesh or muscle) are prerequisites [2,17].

In some studies, a comparative analysis is conducted, evaluating different species, wild versus farmed [19,20,21], young versus elder animals [22,23], geographical [24,25], seasonal variations [26] and environmental versus point-of-sale samples [27].

Wild bivalves have been used as effective biological monitors of marine pollutants due to their sessile nature and large size [20]. Several studies have suggested that MP prevalence is higher in wild bivalves than those collected from freshwater environments [19,20,21]. Areas with more significant human activity and higher population density tend to have significantly higher plastic levels than regions with lower human impact [19]. While plastic concentrations are higher in densely populated areas, environmental factors such as currents, wind, and municipal waste management practices may also influence plastic distribution in less populated regions [20].

The comparative aspect of these studies is crucial for several reasons. First, it sheds light on the differential bioaccumulation of MPs in benthic versus pelagic species, suggesting habitat-specific exposure risks and the influence of feeding behaviors and ecological niches on MP ingestion [28]. Furthermore, geographical variation in MP contamination may reflect regional differences in plastic usage, waste management practices, and oceanic currents, underscoring the global nature of the MP pollution issue. A multi-species approach of MPs analysis in commercially relevant, edible tissues of seafood is suggested for biomonitoring purposes [29]. The significance of studying MPs in the market and point-of-sale samples stems from the direct interface with consumers representing direct entry points for MPs into the diet. Furthermore, market samples can act as indicators of regional pollution patterns and provide insight into regional and consumer variations in exposure levels to MPs [30].

Despite accumulated evidence of MP contamination in different food products, aligning data obtained in different studies is very challenging. The methodologies employed for quantifying MPs are facing technical challenges and require standardization. The heterogeneity of MP types, sizes, and compositions necessitates meticulous methodological rigor to ensure the accuracy and comparability of results across studies. Techniques like Fourier-transform infrared spectroscopy (FTIR) and Raman spectroscopy, each with its strengths and limitations in terms of sensitivity, specificity, and throughput, are becoming a standard in the analysis, as full chemical and morphological characterization of MPs is a prerequisite for risk assessment [31]. The variation in methodological approaches complicates the direct comparison of data across studies [32]. Moreover, data are reported in different units, such as the number of MPs per g or individual or as mass units of MPs/g. Several recent papers highlighted notable disparities in the reported numbers of MPs in foods and beverages, up to two orders of magnitude, warranting a thorough investigation into the factors contributing to these discrepancies [26,33]. Airborne fibers, a frequent contaminant in MP analysis, are frequently discussed, and clean air conditions for MP analysis are strongly recommended [17,34,35]. Multiple factors can contribute to such differences, such as species-specific, seasonal variations, and depuration processes. Still, using different preparation and detection methods also leads to various interpretations.

For instance, our previous study compared the occurrence, size, shape, and polymer type of MPs present in market samples of farmed clams using two widely employed techniques: Nile Red staining (NRS) and microFTIR. The results indicated that insufficient alkaline digestion could overestimate microplastic content when using NRS [22]. Therefore, a standard analysis method is urgently needed to eliminate the inconsistencies between studies and understand the true level of MP contamination in foods and beverages for exposure and further risk assessment to MP. As for seafood, standardized data on the characterization of MPs at the moment are limited at both spatial and temporal scales [32].

The objectives of our study were: (i) to evaluate a highly efficient digestion protocol for isolating MPs from shellfish tissue suitable for the counting of MPs and their complete chemical characterization by microFTIR; (ii) to compare MP contamination of edible parts of various species of frequently consumed commercial crustacean and molluscan (mussels and clams) shellfish collected from markets in different geographic regions (South Korea, Belgium, Serbia, and Croatia); and (iii) to investigate the relationship between MP contamination and species with different feeding patterns and lifestyles, as well as with geographically different point-of-sale markets.

These findings could provide insights into which factors, such as geographical origin or species type, play a more significant role in the presence of microplastics in commercial seafood samples. Consequently, information on the current levels of MP contamination in the edible tissue of commercially purchased shellfish could add to the current understanding of the global spread of MPs, and the direct risk of MPs transfer to humans through consumption.

## 2. Materials and Methods

### 2.1. Reagents, Materials, and Equipment

All chemicals, materials, and equipment used in the study are shown in the Supplementary Materials (Section S1.1, Table S1).

### 2.2. Seafood Samples

All shellfish samples (mussels, clams, and Crustacea shellfish) intended for human consumption were purchased from Korea, Belgium, Croatia, and Serbia fishery markets and/or retail stores. After purchase, samples were immediately stored in ice. Once in the laboratory, animals were wrapped in aluminum foil and stored at −20 °C before the analysis. Individuals from each group of samples were randomly selected for digestion and MPs analysis. Each individual was shelled, and soft tissue was weighed before being placed in a digestion solution. The soft tissue of each individual was used as an individual sample for MPs analysis; therefore, the number of samples for MPs analysis was equal to the number of animals analyzed, in total, 190 individuals. Collected species from the region and the number of each species per region are described in detail in the Supplementary Materials (Section S1.2, Table S2).

### 2.3. Protocol for Isolation of MPs from Shellfish Tissue for microFTIR Analysis

#### 2.3.1. Quality Assurance/Quality Control (QA/QC) of Analyses: External Contamination Controls

All work was conducted using filtered (0.2 μm) Milli-Q water (Smart2Pure 3 UV/UF, Thermo Scientific, Waltham, MA, USA). Before use, all working solutions were filtered through a 0.45 μm PTFE membrane filter. Glassware (Erlenmeyer flasks, graduated cylinders, vacuum filtration sets, Petri dishes, centrifugation tubes, beakers, glass pipettes) and tools (scalpels, tweezers, spatulas, stainless steel filters) were rinsed with Milli-Q water 3 times before use as well as magnetic stirring bars custom made coated by the glass. Stringent measures were implemented to minimize external contamination: experiments were carried out at designated spaces with the digestion and extraction steps conducted in a laminar flow cabinet. Working in a plastic-free, clean room with compatible materials (e.g., glass, inox steel, etc.) and wearing lab coats of suitable fabric (cotton) prevented such potential forms of contamination. Nitrile gloves were always worn during the experiment. To prevent contamination, all sample preparation, filtration, and glassware cleaning steps were performed inside a laminar flow cabinet (LVAC-K1300, Alpina Konin, Poland). The working table in the laminar flow cabinet was covered with aluminum foil to avoid electrostatic effects. Contamination was also avoided when filters were transferred from the laminar cabinet to the microFTIR laboratory. MicroFTIR characterization was conducted in a dedicated laboratory with positive air pressure to prevent contamination of the external space. Blanks and filtered samples were transferred and covered with clean aluminum foil. To account for any MPs from external pollution, air and procedural blanks were performed during sample laboratory processing. Air blanks were prepared by exposing silicone microFTIR filters in the air during sample pretreatment and polymer identification.

#### 2.3.2. Sample Digestion and Extraction Approach

Four digestion protocols were tested to analyze shellfish samples using the protocol, resulting in the most cost-effective digestion. Firstly, two digestion protocols from the literature were compared before the optimal protocol was established: Alkaline digestion (with 10% KOH, 24 h, 60 °C) [36] and combined enzymatic and alkaline digestion (digestion by pepsin followed by KOH digestion) [37]. Afterward, to make the protocol less costly, a third protocol was tested, which includes reversion of orders of digestive fluids: alkaline digestion with KOH, transfer to stainless filter, followed by enzymatic digestion with pepsin, and transfer to the microFTIR silicone filter. Finally, a fourth protocol was evaluated, which is also based on the reversion of orders of digestive fluids but includes an additional enzymatic step with pancreatin, as well as an additional final step with hydrogen peroxide digestion in the following order: alkaline digestion with KOH, transfer to stainless filter, enzymatic digestion by pepsin, enzymatic digestion by pancreatin, chemical digestion by hydrogen peroxide and transfer to the microFTIR silicone filter.

Only edible parts of bivalves and crustaceans were analyzed for the content of MPs [17]. Details of the protocol are provided in the Supplementary Materials (Section S2).

#### 2.3.3. Evaluation of a Protocol for Isolation MPs from Shellfish Tissue for microFTIR Analysis

The protocol regarding digestion efficiency in species intended for MP analysis, recovery rates, and polymer integrity was evaluated by recording microFTIR spectra. Digestion efficiency (%) was calculated as 100× (initial weight of sample − final weight of sample)/initial weight of sample, as proposed by [38]. The evaluation of digestion efficiency and recovery rates is shown in the Supplementary Materials (Section S2.2, Table S3).

#### 2.3.4. Evaluation of microFTIR Spectra

To investigate whether reagents altered the chemical composition of MP polymers in a way that changed their FTIR spectra, making accurate identification difficult, FTIR spectra of in-house made standard plastic particles (LDPE, HDPE, PP, PVC, PS, PA, and PET) before and after digestion were compared. An in-house IR library was created for each type of standard plastic particle before digestion. At least ten particles of each standard were transferred into an Erlenmeyer and subjected to the proposed digestion protocol. After digestion, the spectra of particles were matched through library search (OMNIC Picta Software, version 1.3) to the respective created library. Average matching rates (%) between FTIR spectra of untreated particles and after their exposure to alkaline/enzyme digestion protocol were determined [39]. The chemical identity (% matching) of each particle of standard FTIR spectra after digestion with their FTIR spectra before digestion is presented in Table S4.

### 2.4. Quantitative Analysis and Polymer Identification with microFTIR Spectroscopy

To determine the number of plastic particles of total extracted particles, the entire filter was screened using a microFTIR Imaging System (Thermo Fisher, Waltham, MA, USA). For each measurement, 16 co-scans at a standard spectral resolution of 8 cm^−1^ and a spatial resolution of 6.25 μm in the 4000 to 750 cm^−1^ range were collected. For all particles regarded as plastic, FTIR MP spectra showed a match of at least 70% with the library spectra (Aldrich Polymers, Aldrich Raman, Hummel Polymer and Additives, Coatings Technology, Industrial Coatings, Polymer Additives, and Plasticizers, Aldrich FTIR Collection Edition, and a custom library of common polymer types made in-house), according to an analysis conducted using OMNIC Picta software. In total, library spectra contain 185,993 spectra and 103 spectra libraries. For the MPs identified with a match close to 70%, additional manual inspection of the spectra was performed to validate identification. For all identified MPs, the size and shape of the two dimensions were determined from the microscopic image generated by microFTIR.

The size of MPs on the filter was measured in the longest dimension (maximum Feret diameter) using the digital ruler of OMNIC Picta software. The spatial resolution was 25 μm, and smaller plastics were also detected and recorded, with a size limit of 10 μm.

Most fibers could be easily distinguished from other particle shapes (e.g., fragments or spheres) by their chemical image, but fibers with a large diameter (>5 μm) or short length (<100 μm) could be misidentified as fragments in the chemical mapping Images.

### 2.5. MPs Profiling in Shellfish (Abundance, Size, and Type of Polymer)

Optimized protocol for isolation MPs from shellfish tissue with microFTIR analysis was applied for quantification and chemical characterization of MPs in shellfish samples (Mollusca: clams and mussels, and Crustacea shellfish) from different market origins (South Korea, Serbia, Croatia, and Belgium). As fresh clams could not be purchased in Belgium, a sample from the Serbian market sample was collected. Seafood bought in Serbia had various origins, which were either specified on the package or not (Supplementary Material, Section S1). The unit MP items/g tissue was used to express the MP concentration relative to the wet weight of the soft tissue in grams, while MP items/ind was used to express the number of MPs per individual animal. The complete inner content of the shell was regarded as tissue (for the bivalve’s soft tissue, including intravalvular liquid).

### 2.6. Statistical Analyses

Non-parametric analyses were conducted, as the data showed a non-normal distribution (Shapiro–Wilk test, *p* < 0.05). The Kruskal–Wallis Test evaluated the species variation of MPs in three shellfish species. Further, to compare the statistical significance of MPs in selected species, the Mann–Whitney U test was conducted. Principal Components Analysis (PCA) for microFTIR was conducted based on major dimension, polymer type, mass, and abundance (MPs/g and MPs/ind) of all samples. Analysis of the relationship between the MPs’ size (length and weight) was conducted using the Spearman Correlation test. All the above tests were performed using statistical software SPSS version 23 and PAST v 3.25.

## 3. Results and Discussion

### 3.1. Evaluation of a Protocol for Isolation MPs from Shellfish Tissue for microFTIR Analysis

To select an optimal protocol for sample digestion, we have analyzed the digestion efficiency of four different protocols: chemical digestion based on 10% KOH [36], enzymatic/alkaline protocol [37], and two modified protocols in this study with the sequence of digestive fluids reverted (chemical digestion first and enzymatic second with the hydrogen peroxide step included for polishing of the extracted MPs), one with pepsin only and another one with pepsin followed by pancreatin with further oxidative step. The protocols regarding digestion efficiency in biological samples selected for analysis in our study (Crustacea shellfish, clams, and mussels) were evaluated. The most efficient protocol was further validated for polymer integrity and recovery after alkaline/enzymatic/peroxide digestion.

#### 3.1.1. Efficiency of Digestion

The digestion efficiency of four digestion approaches was tested on Mediterranean mussel (*Mytilus galloprovincialis*), Vongole clam (*Venerupis decussate*), and Crustacea shellfish (*Nephrops norvegicus*) (Table 1). The obtained efficiency of alkaline digestion is greater than 98%, which agrees with the results of other authors [36]. Digestion efficiency for all tested species using combined alkaline and one enzyme digestion was even higher (greater than 99%). However, a combination of alkaline, two-step enzymatic, and hydrogen peroxide digestion has shown to be superior to the other three tested protocols, resulting in an efficiency of digestion ≥ 99.8% for all three tested species.

**Figure 1:**
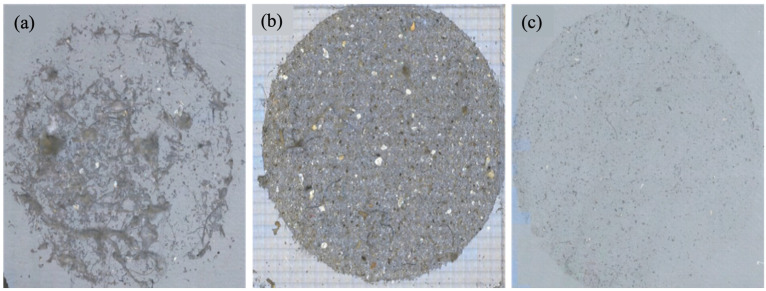
Optical images of the filters obtained after digestion of whole clam individual using different protocols: (**a**) after alkaline digestion protocol [36], (**b**) after enzymatic (pepsin)/alkaline protocol [37] and (**c**) after alkaline/enzymatic (pepsin followed by pancreatin)/oxidative protocol. Optical images of silicone filters with a diameter of 1 cm and pore size of 1 µm were generated by the microFTIR imaging system before analysis by manual and automated methods.

Even though all applied digestion approaches show very high digestion efficiency (above 98%), remaining biological structures and/or remaining material adhered to the MPs when transferred to the silicone microFTIR filter and observed prior MPs identification appear very different (Figure 1). Optical filter images collected after digesting the whole clam using various methods demonstrate how the amount of undigested materials influences the image quality. Although each method has a comparable percentage of digestion efficiency, even minor contaminants clearly affect particle coating.

Oxidative treatment with 15% H_2_O_2_ solution (*v*/*v*) is not suitable for digesting the complete amount of sample material but efficiently removes residues adhering to the filter surface; thus, this step was necessary to remove the remaining biological material present in isolated MP, as confirmed by NR staining of MPs isolated by the third method combined with the final step of H_2_O_2_ oxidative treatment. Fluorescent images of filters after NR staining of MPs isolated by alkaline/enzymatic (pepsin followed by pancreatin) digestion with and without the 15% H_2_O_2_ step are shown in Figure S1 in the Supplementary Material.

Therefore, the alkaline/dual enzyme-pepsin/pancreatin/H_2_O_2_ digestion method (Figure 1c) cleans isolated MPs from the matrix residues efficiently and has a negligible impact on plastic polymers’ integrity. It was selected as the most efficient approach and applied to all samples.

#### 3.1.2. Polymer Recovery and Integrity

Polymer recovery and integrity were investigated, as shown in the Supplementary Material. Recovery rates were in the range of 90 to 100% (Figures S2–S4, Table S4). The integrity of different polymers after digestion was confirmed by microFTIR spectra showing high similarity with a library of spectra (Table S3). The percentages of matching MP spectra of different polymers after digestion with the library spectra were from 75.87–81.04% for PET to 91.23–96.73% for PVC.

#### 3.1.3. External Contamination Controls

Procedural blanks were run with all the reagents without a sample. A total of 17 blank samples were analyzed by microFTIR in the same way as seafood samples (automatic particle recognition and identification). Only seven particles were identified with a match above 60% and manually verified as non-plastic. For the sample’s verification, the threshold for positive identification was set at 70%, and all particles with a match of around 70% were manually examined to confirm identification.

### 3.2. MPs Identification and Quantification in Seafood Samples Collected from Different Markets

#### 3.2.1. Frequency, Abundance, and Species Variation of MPs in Shellfish

MicroFTIR-based counting of particles found in shellfish samples per individual is presented in Table S4. In detail, the counting, identification, and characterization data of MPs in all species from all analyzed regions by microFTIR are presented in Tables S5–S13. Overviews of data of counting, identification, and characterization of MPs in all species from all analyzed regions are presented in Tables S14–S17.

On average, MPs were detected in 43.68% of individuals of all three commercial seafood categories analyzed (clams, mussels, and Crustacea shellfish) with a range of 30% (clams—Korea) to 60% (mussels—Korea and clams—Serbia) (Table 2).

The mean values of the number of MPs/g of wet weight of the edible parts of all animals analyzed, as well as the mean value of the number of MPs/g of wet weight of the edible parts in the samples with MPs found, were presented in Table 2. The average content of MPs (MPs/g tissue) in Crustacea shellfish was the lowest regardless of the origin (Table 2). Detailed results of determined MP content in each sample of clams, mussels, and Crustacea shellfish from South Korea, Croatia, Belgium, and Serbia are provided in Tables S5–S13 (Supplementary Data). Furthermore, an overview of mean values ± SD and range for counting and characterization of MPs in different species from different regions are summarized in Tables S14–S16. The highest MP concentration was four MP particles/individual found in the edible tissue of clams from Korea (Table S5, Supplementary Data).

**Figure 2:**
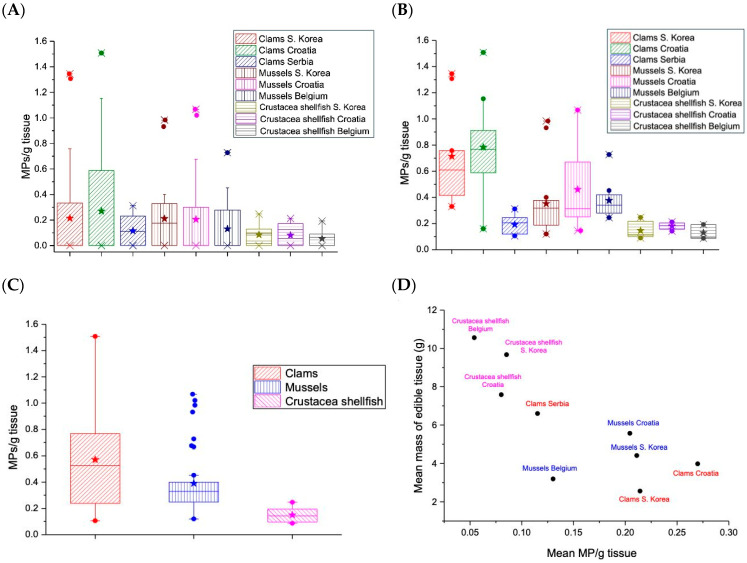
Box plots of the distribution of MP content per g for each species in each country, showing the median, quartiles, and outliers within the data for the whole data set (**A**) and the subset of samples with MPs identified (**B**). For the subset of samples with MPs, all food species were identified and grouped regardless of origin (**C**). Correlation plot between mass of edible tissue and mean MPs abundance in the sample group (MPs/g tissue) (**D**).

No statistical differences (Kruskal–Wallis test, *p* > 0.05) were found when analyzing the whole set of samples according to mean MPs/g tissue between species and origin of seafood (Figure 2A). The mean values of MPs/g tissue for the food category Crustacea shellfish were the lowest across the regions. There was no statistical difference in the estimated frequency of MP occurrences in bivalves collected from geographically distinct markets (Kruskal–Wallis, *p* > 0.05). The mean abundance of MPs in bivalves was not significantly different in locations, calculated either by items per individual or items per gram (Kruskal–Wallis tests, *p* > 0.05). Detailed results of statistical analysis tests are provided in the Supplementary Materials (Table S18).

However, if only a subset of samples with MPs identified was analyzed, there were species-specific statistically significant (Kruskal–Wallis, *p* < 0.05) differences, in particular between clams and Crustacea shellfish (Figure 2B). Detailed results of the statistical analysis tests are provided in the Supplementary Materials (Table S18). When all samples are grouped according to food category (Crustacea shellfish, clams, and mussels) and regardless of market origin, statistically significant differences (Mann–Whitney U test with Bonferroni correction, *p* < 0.05) have been found between all seafood categories analyzed, with the Crustacea shellfish having the lowest abundance of MPs/g (Figure 2C). For all food samples categorized according to nine subgroups (grouped according to market of origin/species), there was a strong negative correlation quotient of 0.79 between the mass of edible tissue and MPs abundance/g tissue (Figure 2D).

**Figure 3:**
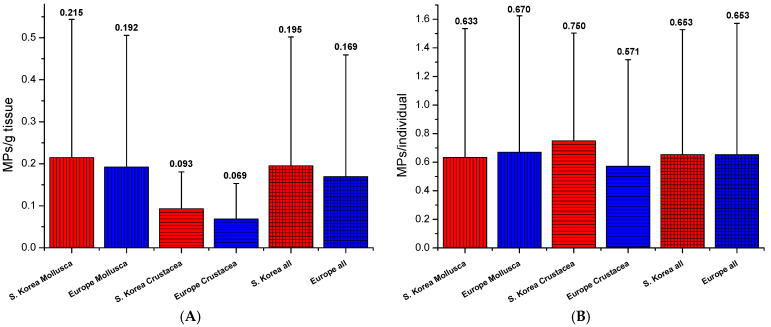
A comparison of the average MP content per gram of edible tissue (**A**) and per individual (**B**) for both non-European (S. Korean) in red and European markets (Belgium, Croatia, and Serbia) in blue for all analyzed samples.

Furthermore, spatial characteristics of MP abundance were considered. The average MPs/g tissue and average MPs/individual of seafood for human consumption were compared for all samples and grouped into a category of non-European and European market origins (Figure 3). No statistical differences were observed for either expression. However, the average MPs/g tissue was slightly higher for the South Korean markets for both food categories (26% for Crustacea shellfish, 11% for clams/mussels), and all seafood samples analyzed (13%). The average MP/individual was higher for European Mollusca (6%) but lower for Crustacea shellfish (24%), and no difference existed for all food samples analyzed.

#### 3.2.2. MP Sizes and Shapes

**Figure 4:**
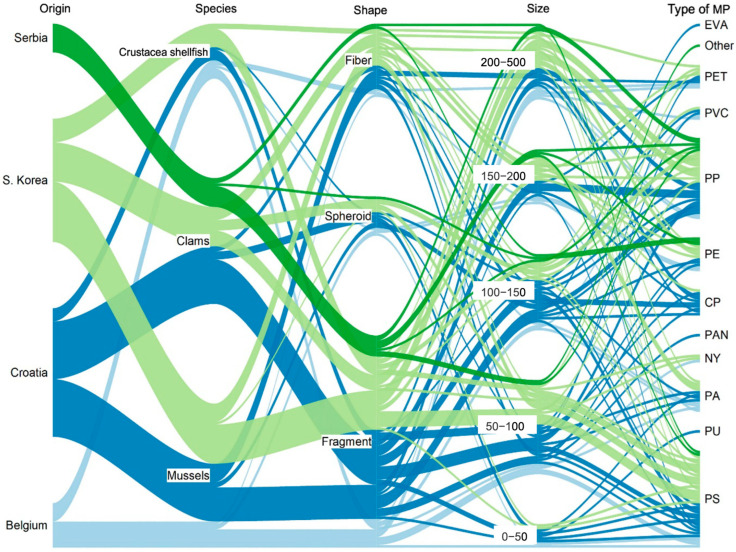
The map of the interrelationship among market location and physicochemical properties of MPs identified in clams, mussels, and Crustacea shellfish (size, polymer type, and shape).

The sizes of MPs found in all three commercial samples were categorized into four groups comprising <50, 50–100, 100–150, 150–200, and >200 μm. The size distribution of MPs in bivalve and Crustacea shellfish samples is shown in Figure 4.

Smaller MPs predominate in all food categories analyzed (size less than 150 μm). The average length ranged from 105.97 to 166.27 μm for clams, 149.14 to 206.91 μm for mussels, and 93.46 to 274.64 μm for Crustacea shellfish. The average width ranged from 50.30 to 80.46 μm for clams, 52.69 to 82.59 μm for mussels, and 59.86 to 95.80 μm for Crustacea shellfish.

**Figure 5:**
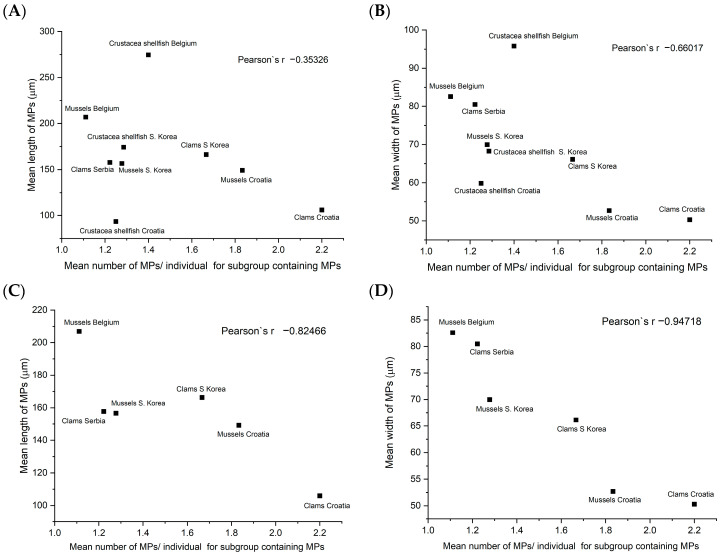
Correlation of mean number of MPs/individual for subgroup containing MPs and MPs length (**A**,**C**) and MPs width (**B**,**D**), for all analyzed species (**A**,**B**) and bivalves only (**C**,**D**).

A negative correlation was also observed between MPs size and the number of MPs/individual for subgroups containing MPs (Figure 5). Although this correlation is relatively poor for all analyzed species (−0.35 for length and −0.66 for width), for bivalves, it is very high (−0.82 for length and −0.95 for width). The correlation of MPs’ size and the number of MPs/g for subgroup containing MPs for all analyzed species is also poor (−0.37 for length and −0.59 for width), while for bivalves, it is higher (−0.50 for length and −0.71 for width). This means that for bivalves, the size of MPs decreases with an increased number of ingested MPs per individual. Figure 5 also shows that the size of MPs is the lowest for all seafood from Croatia and is the highest for all seafood from Belgium. In addition, MPs with lengths > 500 μm were found only in seafood from Belgium (4 of 17 MPs found).

Three different shapes of MPs, including fiber, fragment, and granule (spheroid), were detected in the soft tissue of examined species from all sampling regions (Figure 3). The predominant shape of MPs was fragment (65.3%), followed by fiber (22.6%) and spheroid (12.1%).

The proportions of fragments in clam and mussel samples were in the range of 40–77.27% and 59.09–70.00%, respectively, followed by fibers (9.09–33.33% and 10–31.82%) and granules (9.09–26.67% and 4.35–20.00%), respectively.

#### 3.2.3. Polymer Composition of MPs

**Figure 6:**
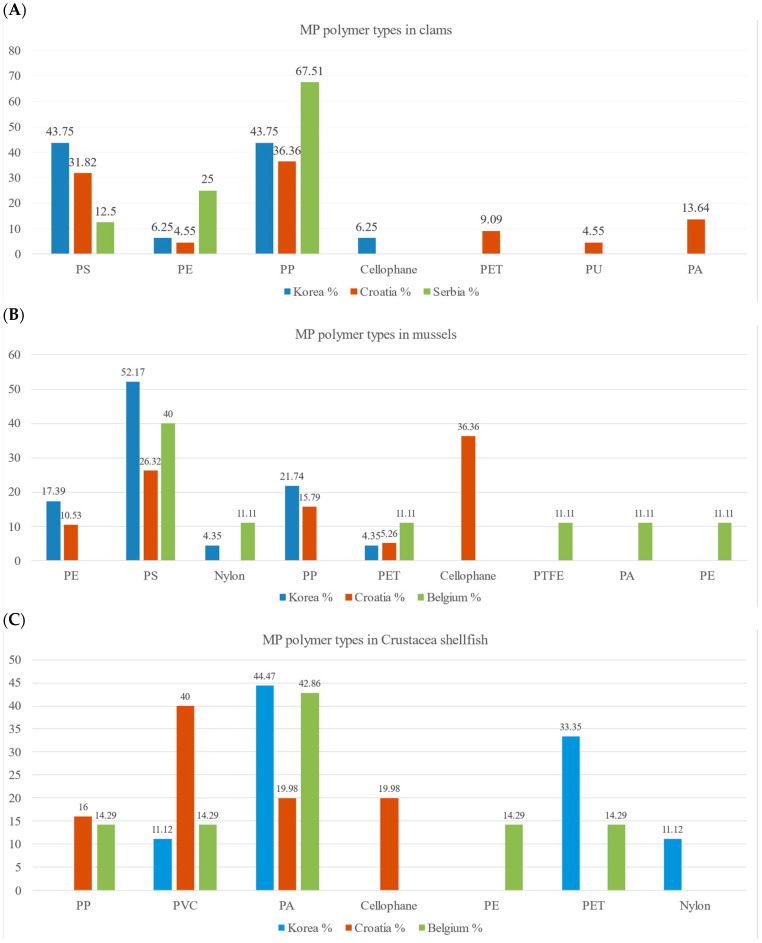
MP polymer types identified in clams (**A**), mussels (**B**), and Crustacea shellfish (**C**) according to the region of origin. The total number is 100% of MP polymer types identified in the sample group (species/region).

The results from the microFTIR analysis of bivalves and Crustacea shellfish samples revealed the presence of more than ten different polymer types of MP, comprising cellophane (CP), polyethylene (PE), polypropylene (PP), polystyrene (PS), polyvinyl chloride (PVC), polyethyleneterephthalate (PET) polyamide (PA), nylon (Ny), polyurethane (PU), polyacrylonitrile (PAN), ethylvynilacetate (EVA) and other types (Figure 4 and Figure 6). The quantities of each type of MP polymer are presented in Table S17. An overview of shapes and types of MPs in different species from different regions is presented in Table S17. PS and PP were the most widely distributed and abundant MP types found in clam samples in two regions, accounting for 86.67% and 68.18% of all found particles in Korea and Croatia, respectively (Figure 6A). PS was the most widely distributed and abundant MP type found in mussel samples in two of three regions of purchase, accounting for 52.17% and 40.00% in Korea and Belgium, respectively (Figure 6B). Cellophane was the most abundant type of mussel in Croatia, accounting for 36.36% of the total (Figure 6B).

Crustacea shellfish from Korea and Belgium were found to have the highest amount of PA (44.44% and 42.86%, respectively), Figure 6C. PET was the second polymer type with 33.33% and 14.29% in Crustacea shellfish from Korea and Belgium, respectively (Figure 6C). Conversely, PVC was the dominant form of MPs in Crustacea shellfish from Croatia (40.0%), followed by PP (16%), Figure 6C. In this study, PAN, EVA, and PU were the least common types of MPs in three commercial seafood samples.

PS, PP, and PE were the most widely distributed and abundant MP types found in bivalves, accounting for 83.33% of MPs in clams and 69.10% in mussels, while PA, PET, and PVC were the most abundant MPs in Crustacea shellfish, accounting for 76.19% of MPs (Figure 6). This indicated that regardless of market of origin, Crustacea shellfish accumulate different types of polymers in comparison to bivalves, and this difference is striking concerning the most abundant polymers (PS and PP) identified in clams and mussels.

#### 3.2.4. PCA Analysis

**Figure 7:**
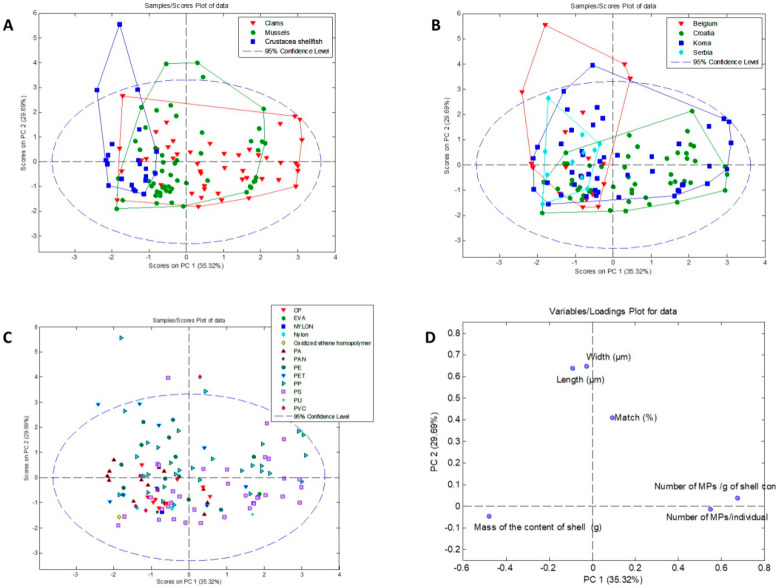
PCA analysis for microFTIR-based on the abundance of MPs (MPs/g), tissue mass, MPs size (length and weight), and density of samples collected from the markets in Korea, Belgium, and Croatia. Score plots (**A**) species trends (clams, mussels, and Crustacea shellfish), (**B**) region trends (Korea, Belgium, Serbia, and Croatia), (**C**) different MP type plot, and (**D**) loading plot describing the model direction/importance of variables.

Principal Component Analysis (PCA) was applied to the results of the abundance of MPs (MPs/g), tissue mass, MP size (length and weight), and density for samples with MP identified, and it was conducted. It resulted in a three-component model, which explained 79.5% of the total variance (Figure 4). The first principal component (PC1) accounted for 35.3% of the overall data variance and 29.7% for the second component (PC2). Considering the PC1 and PC2 score values (Figure 7A), the observed samples formed two distinguished clusters corresponding to Crustacea shellfish and clams, indicating their unique MP accumulation characteristics. On the contrary, the mussel samples were spread on a score plot overlapping with Crustacea shellfish and clamps samples due to their nonspecific accumulation tendency.

Looking for possible patterns between objects according to their geographical origin and MP accumulation tendencies, no trend among the data was observed, i.e., samples from different regions were overlapped (Figure 7B). Similar results were obtained when we observed the influence of polymer type on grouping (Figure 7C). These results indicate that regardless of geographical origin or polymer type, certain species accumulate MPs according to a specific pattern. The most significant influence on clustering had parameters such as the abundance of MPs, tissue mass, and density. In contrast, MP size (length and weight) showed a small contribution (Figure 7D). Figure 7D confirms that tissue mass and number of MPs/g are negatively correlated variables, e.g., the number of MPs/g decreases with the increase in tissue mass. MPs width and length are variables negatively correlated with the number of MPs per individual, suggesting that with an increase of MPs accumulation, their size is smaller.

## 4. Discussion

This study conducted a spatial and inter-species comparison of MP contamination in the edible tissues of commercial shellfish, analyzing samples of Crustacea shellfish, clams, and mussels collected from markets in South Korea, Belgium, Croatia, and Serbia. A unified methodology was employed to assess the frequency of MPs occurrence in individual animals and provide detailed abundance, chemical, and morphological characterization of MPs using microFTIR.

### 4.1. Frequency of MP Contamination

In our study, MPs were detected in 43.68% of the 190 tested individuals, with a 33–60% range in the subgroups analyzed. However, no significant correlation was observed between the frequency of MPs found in analyzed seafood categories regarding the type or origin of samples. Avio et al. [29] suggested the frequency of MPs found in aquatic organisms as a more reliable parameter than MP abundance and a multi-species approach to increase the ecological relevance of assessment and the comparability between different areas and trophic webs. Ingestion of MPs was characterized in 259 organisms belonging to 11 invertebrate species, and MPs were detected in almost all the investigated species, with a frequency of ingestion up to 32%. Nalbone et al. [40] found MPs in 64% of investigated mussels for commercial consumption. In mussels collected in Boka Kotorska, Adriatic Sea, the frequency of MP contamination was 53.3% [41]. It should be noted that composite samples are frequently analyzed. Even in the case of composite samples analysis, MPs were detected in bivalves sold in fishery markets with 95% detection frequency (58 out of 60 composite samples) [30]. In Crustacea shellfish, the frequency of MPs found was in the range of 67% to 80% depending on the species analyzed [42]

### 4.2. Abundance

In animals containing MPs in our study, we have found 0.57 ± 0.40 MPs/g in clams, 0.39 ± 0.25 MPs/g in mussels, and 0.15 ± 0.05 MPs/g in Crustacea shellfish. Due to their wide geographic distribution, sessile lifestyle, and filter-feeding habits, bivalve species have been frequently investigated for MPs content of samples collected from the environment [43]. The abundance of MPs in clams varies from 0.19 ± 0.08 items/g [24] to 4.29 ± 2.09 items/g [44] and from 0.56 ± 0.22 items/individual [24] to 16.6 ± 6.9 items/individual [45]. Similarly, the content of MPs in mussels is in the range from 0.15 ± 0.14 items/g [46] to 3.41 ± 2.97 items/g [47] and from 0.76 ± 0.30 items/individual [24] to 22.26 ± 1.14 items/individual [47]. In shrimps, the content of MPs ranged from 0.04 ± 0.07 item/g [26] to 25.3 items/g of GIT [48] and from 0.39 item/individual for shrimps [26] to 21.8 MP/individual [49]. Therefore, differences higher than two orders of magnitude are found in reported mean abundances (in MPs/g or MPs/individuals) in the literature for the seafood species we have selected to investigate in this study. However, regardless of difficulties attributed to the size detection limit across studies, variations in geographical sampling areas, the use of different protocols and analytical techniques, and the differences in analyzed species, which affect individual body weight and feeding rates, the majority of inter-species comparative studies, as well as reviews summarizing abundance of MPs in bivalves, show higher values of MPs/g in clams in comparison to other seafood [2,28,30].

Furthermore, based on 61 peer-reviewed papers, Ding et al. [28] summarized the current knowledge of MPs in bivalve mollusks globally and found no significant differences in MP abundance among genera from the same family but significant differences among bivalve families, indicating habitats play an essential role in MP ingestion by bivalve mollusks [28]. Our observed concentrations of MPs in bivalves collected from fisherman markets in the Western part of South Korea agree well with the reported abundancy of MPs in commercial bivalves collected from different markets in South Korea [30], both being at the lower range of reported abundance of MPs in bivalves according to the global survey study in environmental samples [28]. The measured abundances of MPs vary with depuration, a process frequently applied for commercial bivalves, and may further be affected by storage and packaging. Cho et al. [50] found that MP abundance was significantly higher in bivalves from Korean coastlines than those purchased from seafood markets. Furthermore, depuration may affect the observed size range of identified MPs. Birnstiel et al. [51] reported that the MP content decreased by 47% after 4 days because of depuration in environmental and farmed mussels, while Van Cauwenberghe and Janssen [35] found a decrease of 30%. For the two species evaluated, the three-day depuration period resulted in the removal of all (in mussels *Mytilus edulis*) or the majority (in oyster *Crassostrea gigas*) of the largest MPs (i.e., particles > 25 μm in length) [35].

Crustacean species are studied less for MPs content, and only a few published studies have reported MPs in shrimps. Hara et al. [52] reported 1.75 ± 2.01 items per individual in shrimps from Irish waters, and Cau et al. [42] 1.66 ± 0.1 to 5.5 ± 0.8 items/per individual in two crustacean species of the Mediterranean Sea. The results could not be compared to ours, as the authors analyzed MPs in the stomachs of shrimps while we studied the MPs content of whole edible tissue of individual animals. In this study, the obtained content of MPs in Crustacea shellfish was in a similar range to those found in shrimps from coastal waters off Cochin, Kerala, India (0.04 ± 0.07 MPs/g) [26]. By contrast, MPs abundance in nine different shrimp species of the Bay of Bengal showed much higher abundance in shrimp gastrointestinal tract and muscles, with the *Caridina cantonensis* exhibiting the highest MPs level with 7.2 items/individual [48].

Asia is known as the largest hot spot of MP pollution, and Lebreton et al. [53] estimated that the amount of plastic debris from Asian countries into the ocean represents 86% of the total global emissions. Reports show the relationship between MPs abundance and MPs abundance in the surrounding water. Despite those reports, in this study, samples from the markets on the Western coast of South Korea (representing samples collected in Asia) in comparison to seafood from the European markets (of various origins) show just slightly higher abundance (20–30%) regarding the average MPs/g for all studied seafood categories.

We have also observed a strong negative correlation between the mean value of MPs/g tissue, an average value of MPs/g tissue in the subgroup of animals that incorporated MP, and maximal MPs/g tissue and the size of the animals (edible parts). This statistical relationship suggests that sample groups made of species of larger animals (by mass of edible tissue) might have lower concentrations of MPs/g of tissue. This strong negative correlation further supports the observation that larger seafood samples (by mass) may contain lower concentrations of MPs on average and at the highest contamination levels.

### 4.3. Size and Morphology of MP

The predominant shapes of MPs in all our analyzed samples were fragments (65.3%), followed by fibers (22.6%) and spheroids (12.1%). Unlike the global survey of MP morphology in environmental bivalves [28], in our study of point-of-sale samples, fragments dominate over fibers in all investigated food groups. In that regard, our data correspond to both environmental and market bivalves of South Korea [30,54], environmental mussels and oyster samples from France [55], and mussels and clams from Portugal [2]. Morphologically, the samples detected in our study of Korean mussels agree with the study of Jeong J et al. [56]. Fragments may occur more frequently in filter feeders, such as clams, as fragments are more widespread in the seawater [57].

Applied for the analysis of 190 individual samples, our methodology allowed to detect and identify MPs that were mostly with size ranging from 50 to 300 μm (predominantly <150 μm), similarly to what has been reported in other studies involving market bivalves of China and South Korea [30,58] and MPs identified globally in bivalves across different studies [28], indicating that small MPs are more likely to be ingested by bivalve mollusks. Most MPs ingested by marine invertebrates and fish are often smaller than 300 μm in diameter [49]. Ding et al. [43] found that the majority of the MPs identified in bivalves in China were smaller than 0.1 mm in size. Even shorter MPs measuring between 0.05 and 0.1 mm were found in mussels collected from the coast of France off the Atlantic Ocean [55]. MP sizes below 250 μm constituted up to 84% of the total MPs in commercial bivalves and 79% in coastal mussels in China [58,59]. The size range of MPs in commercial samples may also be affected by the depuration of particles during transportation and storage, leading to a significant decrease in larger MPs [35]. Our results may also indicate that market samples may have been subject to depuration, providing almost full depletion of larger MPs.

### 4.4. Polymer Identification

We have observed differing polymer compositions according to species. PS was found in high proportions in mussel samples from Korea and Belgium. At the same time, cellophane was the dominant polymer type in mussels from Croatia, with PS being the second most frequently found polymer in Croatia. In clam samples, PP was found to be the more abundant polymer type in all regions investigated (Figure 6). By contrast, in Crustacea shellfish, PS, being dominantly found in mussels and frequent in clams, was not found at all.

In a survey of published data on polymer types of environmental bivalves, PET emerged as the most commonly identified polymer, followed by PE, rayon, PP, cellophane, PES, PA, PVC, PS, and acrylic [28]. PET was particularly prevalent in bivalves from regions such as China, South Africa, Mexico, and Portugal, whereas PE was more dominant in the MP composition of bivalves from Greece, France, New Zealand, and Italy [28]. Given that our samples were sourced from points of sale, they may not reflect the same types of polymers as in environmental studies. MPs may be subject to depuration or be introduced during packaging and transportation.

Studies show that the contamination of MPs in market bivalves may be related to the place where they were cultured [30]. Most oysters, mussels, and scallops sold in the Korean fishery market are cultured on hanging long lines supported by expanded polystyrene (EPS) [30]. Mussels, oysters, and polychaete from three coastal sites representing urban, aquafarm, and rural areas in South Korea were analyzed by Jang et al. [60]. Diverse polymers were found in marine matrices from the urban site, implying diverse MP sources in highly populated and industrialized areas. PS was more abundant in the aquafarm site, reflecting the wide use of expanded PS aquaculture buoys. PP was more abundant at the rural sites, probably due to the use of PP ropes and nets in fishing activities. MP accumulation profiles in marine invertebrates showed trends similar to those exhibited by abiotic matrices, reflecting coastal area use patterns [60]. A study conducted on environmental samples of mussels in Korea showed that PP, PE, and acrylate polymers were dominant MPs [56]. The study also showed that throughout the year, there were only minor variations and no significant monthly changes. In line with these, our findings also highlighted PS, PP, and PE as the second and third most prevalent polymers in Korean mussels, suggesting their association with the aquaculture practices from where these samples originated. Similarly, for plastic materials in blue mussels (*Mytilus edulis*) from the French Atlantic coast, PE and PP fragments were found predominantly [55]. PS was also identified as the primary polymer in point-of-sale Manila clams (*Ruditapes philippinarum*) from South Korea [22]. PP, PS, and PES were identified as the significant polymers in *Mytilus edulis* samples from Korea, aligning with previous reports of market bivalves [30].

In Crustacea shellfish, the ingestion of MPs varies with feeding behavior. For example, the non-selective feeding strategy of *N. norvegicus* may lead to the ingestion of films and fragments from single-use PE and PP products. In contrast, the stomachs of *A. antennatus* shrimp predominantly contained PES filaments, indicating a difference in MPs consumption based on species-specific feeding habits [42]. The primary polymer types detected in shrimps analyzed in Bangalore Bay were PE, PMMA, PVC, PP and Ethylene Vinyl Acetate (EVA) [48]. In our study, PA and PVC could be found in all three groups of Crustacea shellfish collected from markets in Europe and South Korea. *Nephrops norvegicus* (collected from a market in Croatia), Giant tiger prawn (South Korea), and white leg shrimps (from Belgian markets) are all bottom-dwelling crustaceans; therefore, higher density of plastic found in the sample points to the environmental origin of the MPs identified in point-of-sale samples.

### 4.5. Limitations of the Study

Without a unified methodology, comparing results between different studies is a challenge. For instance, not many studies applied microFTIR in reflectance mode and used the whole infrared region for polymer identification. Polymer identification may thus be affected by different spectral ranges covered in our study and represent a methodological bias. A recent study highlighted that sample size, shape, and morphology had a pronounced effect on the reflectance-FTIR signal due to spectral distortion resulting from irregularly shaped particles and non-uniform refractive indices in heterogenous samples [61]. Reflectance mode, however, may create good spectra for thick particles too large to transmit infrared light, providing another methodological bias in our study.

Similarly, FTIR could not create spectra for very thin, colorless fibers [62]. In our study, the samples were collected from various markets (South Korea and Serbia) during the winter season, when the abundance of MPs is anticipated to be at its lowest. Consequently, our findings might not accurately represent the annual average abundance of MPs in seafood globally. Considering the size limitation of the applied methodology (20 μm), smaller sizes of MPs and NP are overlooked in our study and may show very different biodistribution [63]. Despite these limitations, a unified methodology was employed across all samples, offering a snapshot of the potential transfer of MPs of a size larger than 20 μm from various seafood sources.

## 5. Conclusions

In this study, we conducted a comparative analysis of MPs content and types found in three different seafood categories sourced from markets located in geographically diverse areas, encompassing both European and non-European countries. Our investigation involved the application of a standardized methodology for the extraction, identification, and characterization of MPs, which was applied to 190 individually analyzed samples. MPs were identified in 43.3% of individually analyzed animals. Our research into the MP content within the edible tissues of commercially available clams, mussels, and Crustacea shellfish indicated that all samples exhibit a negligible MP content per gram of edible tissue, pointing to depuration strategies applied widely for market seafood. Only in the case of MP-contaminated samples were variations in MP accumulation pertinent to the species observed, with Crustacea shellfish showing the lowest abundance of MPs/g of edible tissue. A negative correlation was identified between the size of the edible tissue of the seafood group and the abundance of MPs, suggesting that the data do not support the bioaccumulation or biomagnification of MPs with the size of the animal in the food group in the case of point-of-sale samples. Even though they do not belong to the same but closely related species, all studied point-of-sale samples behaved similarly and more distinct regarding the food group than the geographical origin of the market. The results of our study do not support significant MP transfer to humans by seafood for point-of-sale samples. Therefore, the screening of other seafood species across regions for a prolonged period, considering the seasonal variation, is crucial to creating a more comprehensive risk assessment framework.

## Figures and Tables

**Table 1:** Digestion efficiency (%) of tested digestion protocols applied on Crustacea shellfish, mussels, and clams using different digestion approaches.

Species	Alkaline Digestion, According to Dehaut et al. (2016) [36]	Enzymatic, Pepsin/Alkaline, According to Süssmann et al. (2021) [37]	Alkaline/One Enzymatic/Pepsin	Alkaline/Two Enzymatic-Pepsin/Pancreatin/Oxidative
Crustacea shellfish	98.90 ± 0.09	99.20 ± 0.10	99.20 ± 0.14	99.96 ± 0.09
Mussels	98.70 ± 0.10	99.20 ± 0.12	99.20 ± 0.11	99.80 ± 0.09
Clams	99.10 ± 0.11	99.00 ± 0.11	99.00 ± 0.11	99.96 ± 0.09

**Table 2:** The number of MPs/g edible tissue found in seafood analyzed in four regions.

Species and Sample Location of Purchase	Number of Individuals Analyzed	Mean Mass of Edible Tissue	% Individuals Containing MPs (Frequency of Ingestion)	Mean Number of MPs/g Edible Tissue +/− SD in All Tested Individuals	Mean Number of MPs g Edible Tissue +/− SD in Individuals Containing MPs	Max Number of MPs/g Edible Tissue Found in Individual
Clams Korea	30	2.56 ± 0.78	30	0.21 ± 0.39	0.71 ± 0.38	1.35
Clams Croatia	29	3.99 ± 1.91	34.48	0.27 ± 0.43	0.78 ± 0.37	1.51
Clams Serbia	15	6.60 ± 1.81	60	0.12 ± 0.11	0.18 ± 0.08	0.31
Mussels Korea	30	4.42 ± 1.76	60	0.21 ± 0.25	0.35 ± 0.24	0.98
Mussels Croatia	27	5.57 ± 3.73	44.44	0.20 ± 0.31	0.46 ± 0.32	1.07
Mussels Belgium	26	3.20 ± 0.95	34.62	0.13 ± 0.20	0.38 ± 0.15	0.73
Crustacea shellfish Korea	12	9.67 ± 1.72	58.33	0.09 ± 0.09	0.15 ± 0.06	0.25
Crustacea shellfish Croatia	9	7.58 ± 3.34	44.44	0.08 ± 0.10	0.18 ± 0.03	0.21
Crustacea shellfish Belgium	12	10.56 ± 1.52	41.67	0.05 ± 0.08	0.13 ± 0.05	0.19

## Data Availability

The data supporting this study’s findings are available through the University of Belgrade—Faculty of Chemistry repository: https://hdl.handle.net/21.15107/rcub_cherry_6483 (accessed on 21 November 2024).

## Supplementary Materials

The following supporting information can be downloaded at: https://www.mdpi.com/article/10.3390/foods13233793/s1. Figure S1. Images of filter after Nile Red staining of digested whole clam individual by alkaline digestion protocol and after alkaline/two enzymatic/oxidative -pepsin/pancreatin protocol. Figure S2. Fluorescent image of silicon filter with 8 Nile red stained particles of standard PVC size 100 µm PP size 500 µm. Figure S3. Representative FTIR spectra of standard particles after complete alkaline/enzyme/oxidative digestion in comparison to spectra from libraries having the highest match. Figure S4. Comparison of FTIR spectra of PET from the library, standard PET particle before digestion, and standard PET particle after complete alkaline/enzyme/oxidative digestion. Table S1. List of in-house made polymer standards used in polymer recovery and integrity testing. Table S2. Selected saefood species and number of samples analyzed per region (Korea, Croatia, Belguim and Serbia). Table S3. Testing of influence of alkaline/enzyme/oxidative digestion on standard particles (LDPE, HDPE, PP, PVC, PS, PA, PET) recovery and integrity. Table S4. microFTIR based counting of particles found in shellfish samples per individual. Table S5–S13. Counting, identification and characterization MPs in clams, mussels and Crustacea shellfish from South Korea, Croatia, Belgium and Serbia by microFTIR. Table S14. Overview of counting of MPs in different species from different regions. Table S15. Overview of mean values ± SD for counting and characterization of MPs in different species from different regions. Table S16. Overview of ranges for counting and characterization of MPs in different species from different regions. Table S17. Overview of shapes and types of MPs in different species from different regions. Table S18. The significance of microplastic transfer interactions for MP abundance of studied species according to statistical analyses.
