# Supplementary material for: The Global Spread of Microplastics: Contamination in Mussels, Clams, and Crustaceans from World Markets
Source: Foods. 2024 Nov 26;13(23):3793. doi: 10.3390/foods13233793 (PMC11640221; doi:10.3390/foods13233793)
Supplement: Supplementary file 1 [file foods-13-03793-s001.zip › foods-3268927-supplementary.pdf]

Supplementary material for

The Global Spread of Microplastics: Contamination in Mussels, Clams, and Crustaceans from World Markets

Tamara Mutić[1], Jelena Mutić[1], Miloš Ilić[1], Vesna Jovanović[1], Jelena Aćimović[1], Boban Andjelković[1], Dragana Stanić-Vučinić[1], Maria Krishna de Guzman[2,3], Mirjana Andjelkovic[3], Mirjana Turkalj[4,5,6], and Tanja Cirkovic Velickovic[1,7*]

[1] Center of Excellence for Molecular Food Sciences, University of Belgrade - Faculty of Chemistry, Studentski trg 12-16, 11000 Belgrade, Serbia

[2] Ghent University Global Campus, Center for Food Biotechnology and Microbiology, 21985 Incheon, South Korea

[3] Department of Food Technology, Safety and Health, Faculty of Bioscience Engineering, Ghent University, B-9000Ghent, Belgium

[4]Srebrnjak Children's Hospital, HR-10000 Zagreb, Croatia

[5]Faculty of Medicine, J.J. Strossmayer University of Osijek, HR-31000 Osijek, Croatia

[6] School of Medicine, Catholic University of Croatia, HR-10000 Zagreb, Croatia

[7]Serbian Academy of Sciences and Arts, Knez Mihajlova 35, 11000 Belgrade, Serbia

Content of Supplementary material:

Tables:

Table S1. List of in-house made polymer standards used in polymer recovery and integrity testing

Table S2. Selected saefood species and number of samples analyzed per region (Korea, Croatia, Belguim and Serbia)

Table S3. Testing of influence of alkaline/enzyme /oxidative digestion on standard particles (LDPE, HDPE, PP, PVC, PS, PA, PET) recovery and integrity.

Table S4. microFTIR based counting of particles found in shellfish samples per individual.

Table S5-S13. Counting, identification and characterization MPs in clams, mussels and Crustacea shellfish from South Korea, Croatia, Belgium and Serbia by microFTIR.

Table S14. Overview of counting of MPs in different species from different regions.

Table S15. Overview of mean values ± SD for counting and characterization of MPs in different species from different regions.

Table S16. Overview of ranges for counting and characterization of MPs in different species from different regions.

Table S17. Overview of shapes and types of MPs in different species from different regions.

Table S18. The significance of microplastic transfer interactions for MP abundance of studied species according to statistical analyses.

Figures:

Figure S1. Images of filter after Nile Red staining of digested whole clam individual by alkaline digestion protocol and after alkaline/two enzymatic /oxidative -pepsin/pancreatin protocol.

Figures S2. Fluorescent image of silicon filter with 8 Nile red stained particles of standard PVC size 100 μm PP size 500 μm.

Figure S3. Representative FTIR spectra of standard particles after complete alkaline/enzyme /oxidative digestion in comparison to spectra from libraries having the highest match

Figure S4. Comparison of FTIR spectra of PET from the library, standard PET particle before digestion, and standard PET particle after complete alkaline/enzyme /oxidative digestion.

Section S1 Materials

S1.1 Chemicals, reagents and equipment

Chemicals: KOH (Merck, Darmstadt, Germany), Pepsin from porcine gastric mucosa, lyophilized powder 2500 units/mg (Sigma Adrich, St. Louis, USA), Pancreatin from porcine pancreas (4 USP specifications, Sigma Adrich, St. Louis, USA), $NaHCO_3$ (Merck, Darmstadt, Germany), abs EtOH (HPLC grade, Merck, Darmstadt, Germany) and H_2O_2 (30%, Merck, Darmstadt, Germany) were used for organic matter digestion and isolation of MPs. Nile red dye (NR) (Tokyo Chemical Industry Co., N0659, Tokyo, Japan) was used for fluorescent tagging of MPs. Enzyme solutions for digestion were prepared daily according to the procedures described below.

Filters: GF/A glass microfiber filters (Whatman, 1820-047, 1.6 µm, Sigma Adrich, St. Louis, USA), PTFE membrane filter (0.45 µm, (Hyundai Micro,Seoul, Korea) and stainless steel filters pore size 20 and 10 µm (Xinmingde Machinery, Henan, China) were used for filtration.

The silicon filter, 1.0 x 1.0 cm, pore size 1 µm (ThermoFisher, Waltham, USA) was used for filtration prior MPs identification. Silicon filters were used with the FTIR microscope in reflection analysis, facilitating the examination of specific spectral ranges critical for MPs identification. They offer a broader spectral range, allowing for a more comprehensive analysis of MPs.

Instruments: MicroFTIR (microFTIR, iN10 Nicolet, Thermo Fisher scientific, Waltham, USA) which couples a microscope with an infrared spectrophotometer was used for MPs identification. The instrument is equipped with an ultra-fast motorized stage and a liquid nitrogen cooled mercury cadmium telluride detector (MCT detector). Fluorescence microscope (Olympus SZX10 stereomicroscope, Olympus, Tokyo, Japan) equipped with SZX2-FGFPHQ filter set for the excitation from 460 to 480 nm and emission from 495 to 540 nm was utilized for visualization of fluorescently stained MPs.

Standards: Seven in-house produced MP standards (50-500 µm) were used: polypropylene (PP), high-density polyethylene (HDPE), low-density polyethylene (LDPE), polyvinylchloride (PVC), polystyrene (PS), polyethylene terephthalate (PET), polycaprolactam (PA-6), were obtained from Ghent University Polymer Department and characterized as described previously [1]. List of polymer standards with their average size and density is presented in Supplementary Table S1.

Monodisperse microparticle size PS standard dispersion (Supelco, Product No 59336, approximately $3.6x10^4$ particles/ml, of uniform size 100 µm) was purchased from Sigma-Aldrich (Sigma Adrich, St. Louis, USA).

Table S1. List of in-house made polymer standards used in polymer recovery and integrity testing

No	Polymer	Name	Size (µm diameter)	Density (g/ml)
1	PP	Polypropylene	500	0.89-0.91
2	HDPE	High density polyethylene	500	0.95-0.97
3	LDPE	Low density polyethylene	500	0.91-0.94
4	PVC	Polyvinyl Chloride	100	1.20-1.45
5	PS	Polystyrene	120	0.94-1.11
6	PET	Polyethylene terephthalate	120	1.38-1.39
7	PA6	polycaprolactam	120	1.13-1.5

S1.2. Seafood samples

Five to fifteen kg per sample of selected species intended for human consumption was purchased from fishery markets in Korea, Belgium and Croatia. Manila clams (*Venerupis philippinarum*), Korean mussel (*Mytilus coruscus*) and white leg shrimp (*Litopenaeus vannamei*, synonym *Penaeus vannamei*) from Korea were purchased fresh in fish markets in December 2022 (Oido near Siheung, and Soraepogu, Incheon, traditional fish markets) near Western Korean cost. From purchased shellfish quantity of each of the subgroup (species/origin) between 9 and 30 individual animals were randomly selected for digestion and MPs analysis in order to get representative sample for each subgroup. All samples from Belgium, blue mussel (*Mytilus edulis*) and Black tiger shrimp (*Penaeus monodon*), were purchased packed as frozen at several large retail markets in Brussel and Ghent during June 2022. Fresh/frozen clams could not be purchased in Belgium in the period when the collection was made. All samples from Croatia, mediterranean mussel (*Mytilus galloprovincialis*), Vongole (*Venerupis decussate*) and Scampi (*Nephrops norvegicus* .), were purchased fresh during April 2022 from traditional fish markets in Dalmacija region (Croatia). Clams (Vongole, *Venerupis decussate*) from large retail markets in Belgrade (Serbia) were purchased fresh in December 2022. After purchase, samples were immediately stored in ice. Once in the laboratory, animals were wrapped in aluminum foil and stored at −20 °C prior to the analysis. Collected species from region together with number of each species per region is presented in Table S2 (Supplementary material).

Each mussel (average mass of soft tissue with intravalvular liquid was 4.41 ± 2.59 g) and clam (average mass of soft tissue with intravalvular liquid was 3.94 ± 2.12 g) was shelled, and weighed before being placed in digestion solution. Crustacea shellfish were rinsed and carefully shelled. The Crustacea shellfish tissue was weighed (average mass of soft tissue was 9.42 ± 2.46 g) before being placed in digestion solution. The whole soft tissue of each bivalve, hereafter indicated as a sample, was used as an individual sample for MP analysis, therefore the number of samples for MP analysis was equal to the number of animals analyzed, in total 190 individuals.

Table S2. Selected saefood species and number of samples analyzed per region (Korea, Croatia, Belguim and Serbia)

	Clams Venerupis spp.	Mussels Mytilus spp.	Crustacea shellfish
Korea	Manila clam (*Venerupis philippinarum*)<br>n= 30<br>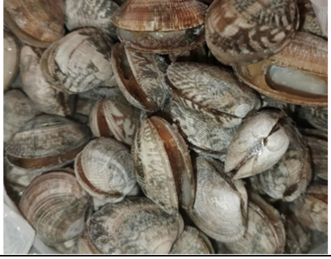	Korean mussel *(Mytilus coruscus)*<br>n= 30<br>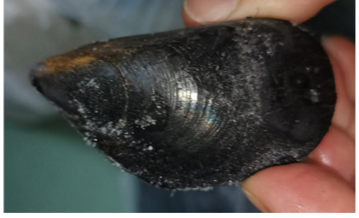	Whiteleg shrimp<br>*Litopenaeus vannamei,*<br>synonym *Penaeus vannamei)*<br>n= 12<br>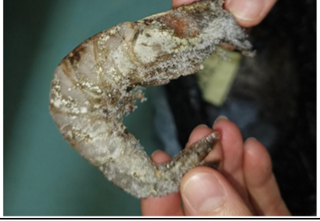

Croatia	Vongole (*Venerupis decussate)*<br>n= 29<br>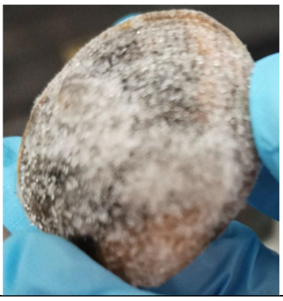	*Mediterranean mussel (Mytilus galloprovincialis)*<br>n= 27<br>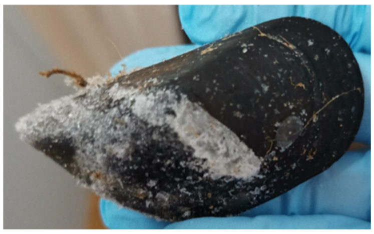	*Nephrops norvegicus*<br>n= 9<br>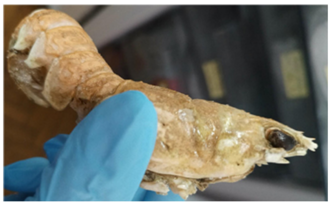
Belgium	*Not found on the market*	Blue mussel (*Mytilus edulis)*<br>n= 26<br>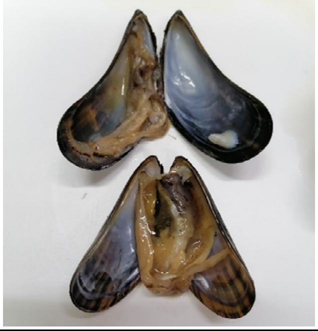	Black tiger shrimp *(Penaeus monodon)*<br>n= 12<br>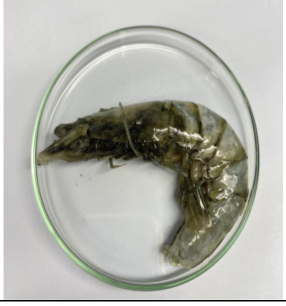
Serbia	Vongole (Venerupis decussate *.)*<br>n=15<br>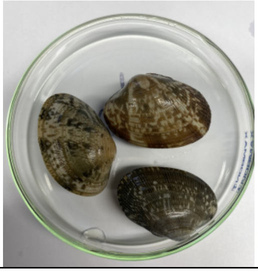	*Not collected*	*Not collected*

Section S2 Validation of protocol for isolation of MPs from seafood samples

S2.1 Efficiency of digestion

Protocol of chemical/enzymatic digestion of seafood samples

Frozen whole content of shell of bivalves (soft tissue with intravalvular liquid) was excised from the shell using a scalpel, weighed, and transferred to an Erlenmeyer flask containing 150 mL of 10% (w/w) KOH solution. Digestion was done at 60 °C, with rotation speed set at 200 rpm for 24 hrs once the temperature was reached. Each individual animal was digested separately and thus was placed in separate digestion flask. After 24 hrs each sample was vacuum filtered through a stainless-steel filter (mesh size 10 µm, 25 mm diameter) and rinsed well with warm milliQ water (60ºC), using a maximum 200 mL of water. The filter was returned in the same Erlenmeyer flask totally covered with 20 mL of pepsin solution (10 IU/mL) and stirred 200 rpm for 2 hrs at 37ºC. Pepsin solution was prepared by dissolving 1 mg of pepsin (2500 U/mg) in 250 mL of 0.063 mol/L HCl and filtered through 0.45 µm. Pepsin solution was prepared daily prior to digestion.

After two hrs, 20 mL of pancreatin solution was added and digestion was continued for the next 2 hrs at 37ºC with stirring. Pancreatin solution was prepared by suspending 1 g of pancreatin (4 x USP) with 300 mL $NaHCO_3$ (0.75 mol/L; pH 8.9). After centrifugation 5 minutes at 5000 rpm, the supernatant was initially filtered through a 10 µm stainless steel filter, and finally through a 1.6 µm glass fiber filter (47 mm). Once the enzymatic digestion was over, the solution was filtered through a new 10 µm stainless steel filter. The Erlenmeyer flask and filter were rinsed with a maximum of 100 mL of MilliQ water. To further reduce residues of the sample matrix the stainless-steel filter was left at room temperature during 16 hrs fully covered in 15 mL of 15 % H_2O_2 solution (v/v).

Transfer to microFTIR filter

The final step of protocol was transfer of isolated MPs from stainless steel filter onto silicone microFTIR filter (1 µm). The stainless-steel filter with MPs was put in beaker of 100 mL where it was rinsed several times with the 50% (v/v) ethanol solution. Before this step, the silicon filter for microFTIR was rinsed several times with 50 mL of ethanol 50% (v/v). After filtration of isolated MPs, the silicon filter was carefully removed from the filtration system, put in a labeled, covered petri dish, and left to dry at room temperature prior the analysis on microFTIR.

Evaluation of digestion efficiency

Digestion efficiency of the optimized protocol was tested regarding its suitability for application on all collected species (clams, mussels, Crustacea shellfish). The digestion efficiency was expressed in a percentage as the ratio between matrix residue after digestion and sample weight before digestion. The weight of the matrix residue was determined by the weight difference of the dried filter (GF 1.6 µm). Dry weight of each filter, obtained by placing filters at 60 ^{0}C for 24 h, was measured before and after digestion to assess the proportion of remaining organic matter on each filter after digestion.

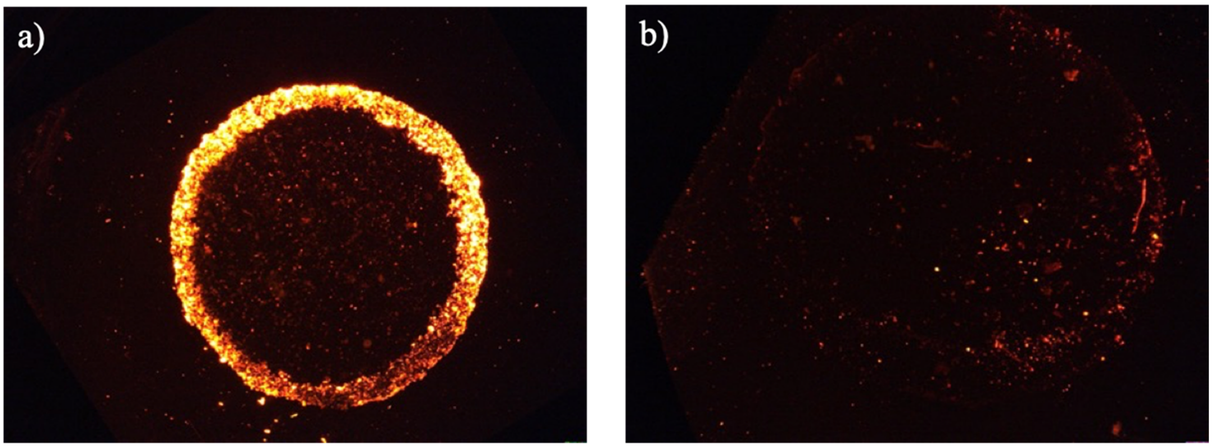

Figure S1. Fluorescent images of silicon filter after Nile Red staining of digested whole clam individual by alkaline digestion protocol [2] (**a**) and after alkaline/two enzymatic (pepsin followed by pancreatin)/oxidative - pepsin/pancreatin protocol .

S2.2. Recovery of MPs particles subjected to developed protocol for digestion

Evaluation of recovery rates

To evaluate our developed protocol for digestion, the recovery (%) of MP particles after digestion was determined both by NR staining and by microFTIR analysis. Particle counting was performed on a fluorescent microscope and with microFTIR. Seven polymer types (LDPE, HDPE, PP, PVC, PS, PA, PET) were stained with NR before digestion. NR solution was in acetone at a final concentration of 10 μg/mL. Staining was performed for 30 min at 60 °C with constant shaking. The dyed MPs were filtered using 25 mm GF/A filter (1.6 μm pore size). The filter was washed with 50 mL absolute ethanol to remove excess dye and reduce background fluorescence. At least ten particles of each standard were observed and counted with a fluorescence microscope. The stained particles were transferred into an Erlenmeyer and treated with the digestion reagents according to protocol (Section 2.3.3.). After digestion, the particles were transferred to filter for microFTIR and rinsed with MiliQ water. All filters with standards were analyzed with a fluorescence microscope and all images processed by software (ImageJ). A recovery percentage was calculated as a ratio of the count of particles lying on filters after digestion and the number of particles subjected to digestion for each polymer type.

The method recovery was evaluated using in-house made standards of following polymer types: LDPE, HDPE, PP, PVC, PS, PA, PET (Their characteristics are provided in the Table S1. Standard particles were stained by Nile Red and counted by fluorescent microscope. Counted standard particles were subjected to complete alkaline/enzyme (pepsin followed by pancreatin)/oxidative digestion procedure and finnaly again counted by fluorescent microscope. A recovery rates (%) was calculated as a ratio of the number of particles counted after digestion and the number of particles counted before digestion . Recovery rates were in range from 90 to 100 % (Table S3).

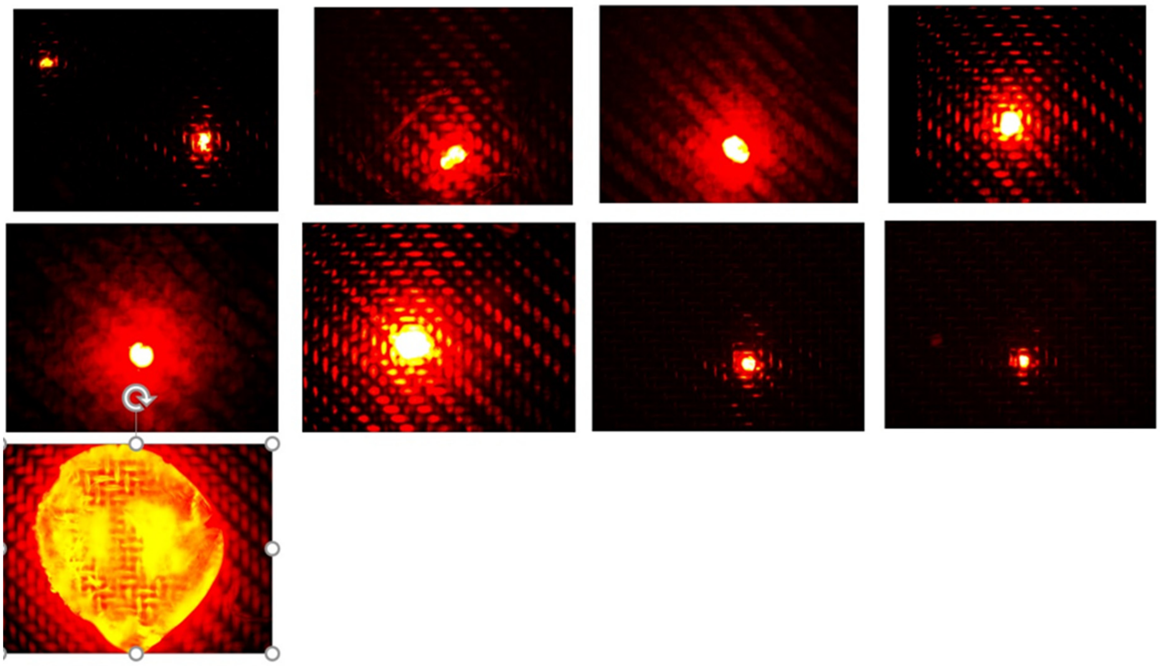

Figure S2. Fluorescent image of silicon filter with 8 Nile red stained particles of standard PVC (polyvinyl chloride) size 100 µm (the first two rows, one image for each particle), and one Nile red stained particle of standard PP (polypropylene) size 500 µm (the third row).

S2.1.1. The integrity of MPs standards after digestion

To check the influence of the optimized alkaline/enzyme (pepsin followed by pancreatin)/oxidative digestion on the integrity of MPs, chemical identification of standard particles (LDPE, HDPE, PP, PVC, PS, PA, PET) was performed before and after complete digestion procedure by micro-FTIR. Beside, FTIR spectra of standard particles after digestion were compared with spectra libraries (Figures S3 and S4).

Figure S3. Spectra of standard particles after complete alkaline/enzyme (pepsin followed by pancreatin)/oxidative digestion in comparison to spectra from libraries having the highest match: (a) PET, b) PVC, c) PP

a)

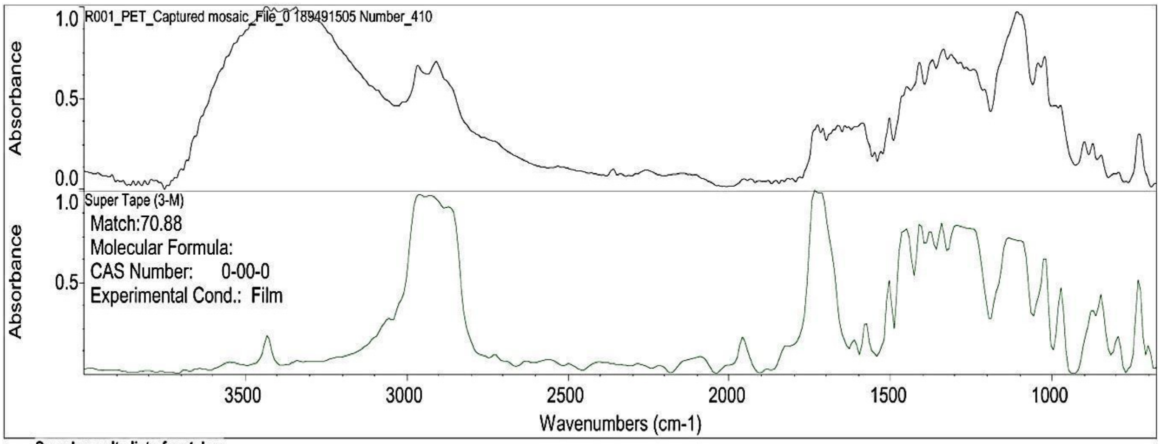

Search results list of matches

	Index	Match	Compound Name	Library Name
1	3527	70.88	Super Tape (3-M)	Toronto Forensic
2	242	69.20	POLYESTER	Synthetic Fibers by Microscope
3	275	67.94	POLYESTER	Synthetic Fibers by Microscope
4	236	67.27	POLYESTER	Synthetic Fibers by Microscope
5	241	67.06	POLYESTER	Synthetic Fibers by Microscope

b)

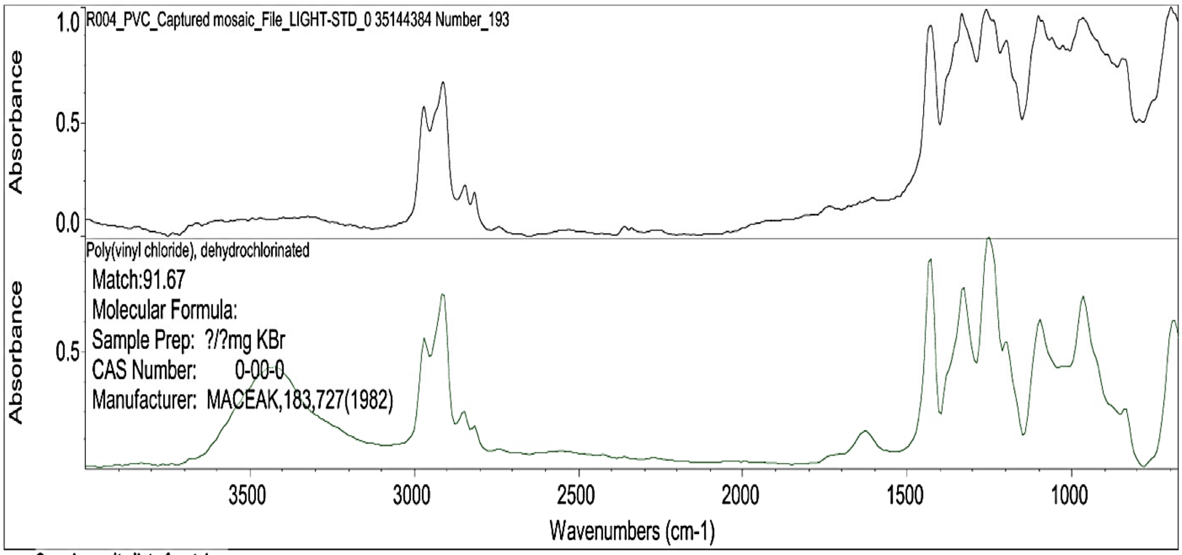

Search results list of matches

	Index	Match	Compound Name	Library Name
1	941	91.67	Poly(vinyl chloride), dehydrochlorinated	Hummel Polymer and Additives
2	941	89.00	Poly(vinyl chloride), dehydrochlorinated	HR Hummel Polymer and Additives
3	1435	81.85	GEON 137X574	Industrial Coatings
4	241	79.95	Poly(vinyl chloride)	Hummel Polymer and Additives
5	989	79.94	Poly(vinyl chloride)	Nicolet Condensed Phase Academic Sampler

c)

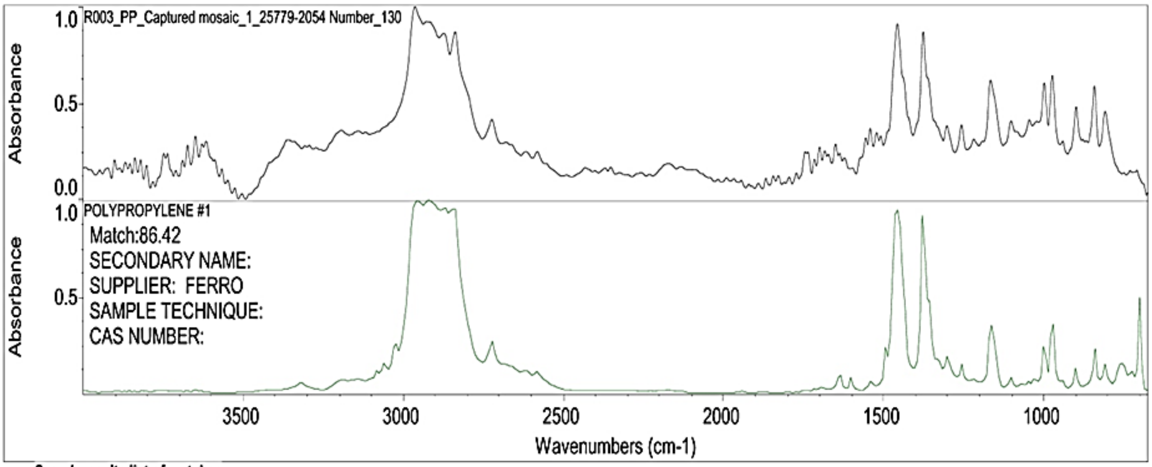

Search results list of matches

	Index	Match	Compound Name	Library Name
1	328	86.42	POLYPROPYLENE #1	Polymer Additives and Plasticizers
2	150	82.26	OLEFIN	Synthetic Fibers by Microscope
3	57	81.82	POLYPROPYLENE HOMOPOLYMER #1	Polymer Additives and Plasticizers
4	328	80.50	POLYPROPYLENE #1	HR Polymer Additives and Plasticizers
5	145	80.47	OLEFIN	Synthetic Fibers by Microscope

Figure S3. Representative FTIR spectra of standard particles after complete alkaline/enzyme (pepsin followed by pancreatin)/oxidative digestion in comparison to spectra from libraries having the highest match: (a) PET, b) PVC, c) PP. Under each pair of spectra the list of 5 spectra from libraries, having the highest matches with standard particles after digestion, are presented.

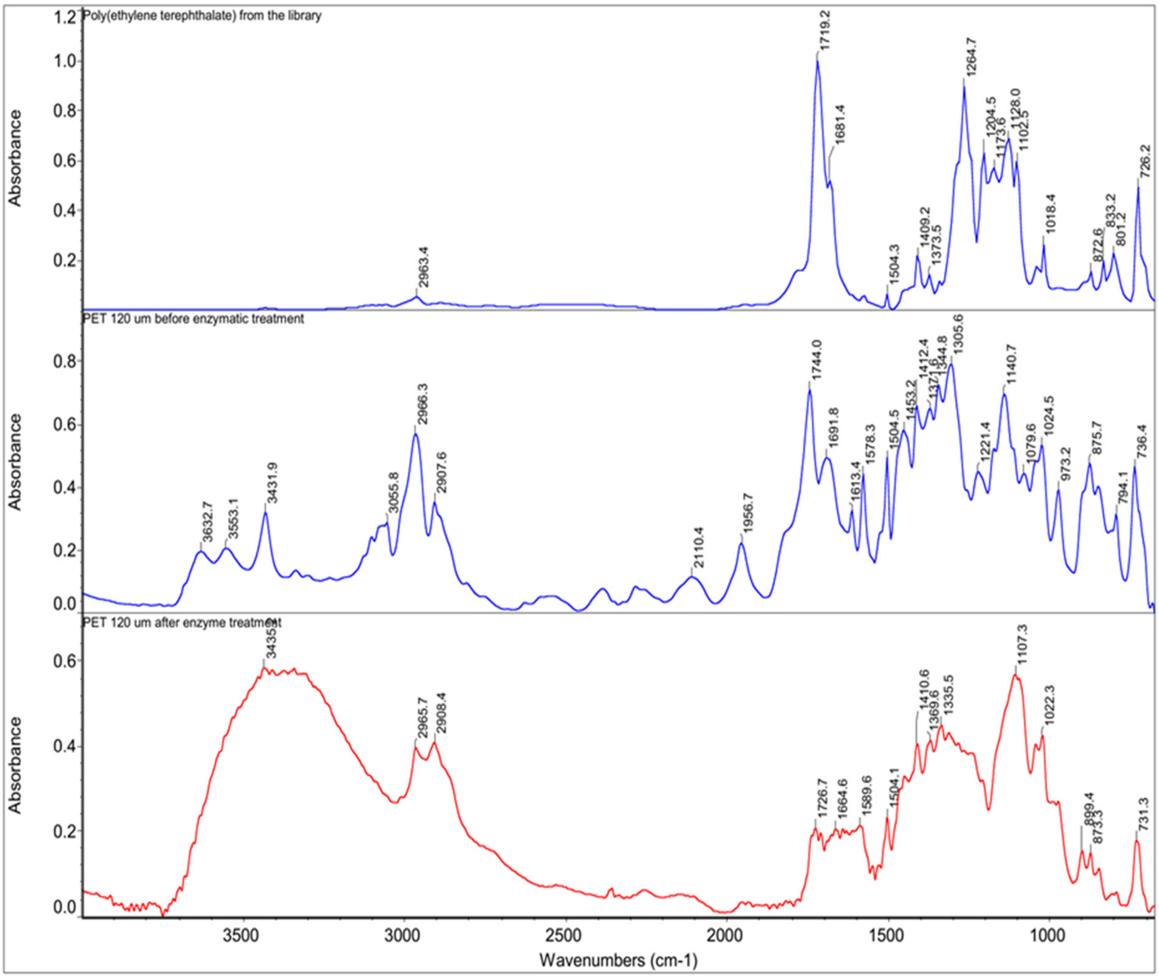

Figure S4 Comparison of FTIR spectra of PET from the library (upper panel), standard PET particle before digestion (middle panel), and standard PET particle after complete alkaline/enzyme (pepsin followed by pancreatin)/oxidative digestion (bottom panel).

As expected, 24 hrs alkaline treatment, followed by a mild enzymatic digestion and peroxide digestion, did not interfere with the chemical identification of seven polymer standards tested in the study (Table S3), according to the criteria set up for a successful identification (>70 % matching). The percentages of FTIR specta matching for different polymers after digestion with the library spectra were from 78.36 (PET) to 94.07 (PVC)%.

Table S3. Testing of influence of alkaline/enzyme (pepsin followed by pancreatin)/oxidative digestion on standard particles (LDPE, HDPE, PP, PVC, PS, PA, PET) recovery and integrity. Integrity of particles was estimated by % of mathing of FTIR spectra of standard particles after digstion and spectra from libraries.

Polymer/Size (µm)	Recovery rate %	% of mathing of FTIR spectra of standard particles after digestion and spectra from libraries

LDPE/HDPE/500	95 ± 5	90.97 ± 3.97
PP/500	98 ± 2	88.81 ± 5.34
PVC/100	85 ± 15	94.07 ± 4.03
PS/120	86 ± 14	79.99 ± 3.98
PA/120	80 ± 20	78.38 ± 4.31
PET/120	81 ± 19	78.36 ± 3.27

Section S3 MicroFTIR based identification and quantification of MPs in shellfich samples

Table S4. summarized results of counting of particles found in shellfish samples per individual: average total number of found particles, average number of MPs when identification with match higher than 60% was taken into account, and the range of number of MPs when identification with match higher than 70% was taken into account and manually confirmed. About 1000 of particles were detected onto micro-FTIR filters originating from one individual, but most of these particles were not identified as MPs. Particles which are not identified as MPs are eather non-plastic, or plastic particles with small width (<20 μm, particularly fibers) and/or shape and morphology that does not allow their an identification by microFTIR in reflection mode. The smallest size of particle that could be identified using microFTIR in the reflection mode under manual inspection was 20 μm. Manual inspection of spectra was done for each sample if it had shown more than 60% match with the library. It was a necessary step for particles with 60 - 70% match. For identification of numerous non-MPs particles (organic and inorganic) extended time is necessry. However, the most of non-plastic particles originate most likely from undigested biological material, despite of very high digestion efficiency Even with digestion efficiency of 99.96 % (for clams and Crustacea shellfish) significant mass of undigested material remains on filter. For example, if individual of 5 g was digested with efficiency of 99.96 % , 2 mg of undigested material remains on filter, which is few order of magnitude higher mass than mass of MPs found on filter.

Table S4. microFTIR based counting of particles found in shellfish samples per individual.

		Average total number of target particles per individual (Non-plastic and plastic)	Average number of plastic microparticles per individual identified by micro-FTIR with match > 60%	The range of number of MPs per individual when MPs identification with match > 70 % and manually **confirmed** was taken into account
	S. Korea (n=12)	973 ± 113	6 ± 4	0-2

Crustacea shellfish	Croatia (n=9)	1211 ± 203	8 ± 5	0-2
	Belgium (n=12)	890 ± 177	9 ± 5	0-2
Clams	S. Korea (n=30)	1003 ± 244	8 ± 4	0-4
	Croatia (n=29)	1029 ± 238	9 ± 7	0-3
	Serbia (n=15)	1107 ± 204	11 ± 4	0-4
Mussels	S. Korea (n=30)	995 ± 182	12 ± 5	0-3
	Croatia (n=27)	1156 ± 256	9 ± 4	0-3
	Belgium (n=26)	1097 ± 263	24 ±10	0-2

Table S5. Counting, identification and and characterization MPs in **clams from South Korea** by microFTIR. # - mass of soft tissue + intravalvular liquid mass (g). Polymer abbreviations: cellophane (CP), polyethylene (PE), polypropylene (PP), polystyrene (PS), polyvinyl chloride (PVC), polyethylene-terephthalate (PET) polyamide (PA), and nylon (Ny), polyurethane (PU), polyacrylonitrile (PAN), ethylvynilacetate (EVA).

**Sample name**	**Mass of the content of shell (g)**	**No. of MPs /individual**	**No. of MPs /g of shell content**	**Type of MP**	**Match (%)**	**Length (µm)**	**Width (µm)**	**Shape**
WP4_KOR_V1_F_1	2.24	0	0.00	/				
WP4_KOR_V1_F_2	3.34	0	0.00	/				
WP4_KOR_V1_F_3	1.99	0	0.00	/				
WP4_KOR_V1_F_4	2.99	0	0.00	/				
WP4_KOR_V1_F_5	1.87	0	0.00	/				
WP4_KOR_V1_F_6	2.91	0	0.00	/				
WP4_KOR_V1_F_7	2.87	0	0.00	/				
WP4_KOR_V1_F_8	2.16	0	0.00	/				
WP4_KOR_V1_F_9	2.64	2	0.76	PS	89.65	80.2	41.5	Fragment
					89.99	91.3	76.4	Spheroid
WP4_KOR_V1_F_10	2.79	0	0.00	/				
WP4_KOR_V1_F_11	3.43	0	0.00	/				

WP4_KOR_V1_F_12	3.00	1	0.33	PE	90.25	285.7	94.2	Fragment
WP4_KOR_V1_F_13	3.03	1	0.33	PP	91.54	110.3	51.9	Fragment
WP4_KOR_V1_F_14	3.10	0	0.00	/				
WP4_KOR_V1_F_15	3.12	0	0.00	/				
WP4_KOR_V1_F_16	3.95	0	0.00	/				
WP4_KOR_V1_F_17	2.12	0	0.00	/				
WP4_KOR_V1_F_18	1.35	1	0.74	Cellophane	75.46	155.4	54.4	Fiber
WP4_KOR_V1_F_19	1.64	1	0.61	PP	79.71	213.7	72.1	Fragment
WP4_KOR_V1_F_20	3.06	4	1.31	PP	98.09	229.7	91.2	Fragment
					95.62	130.9	79.1	Spheroid
					92.4	322.2	86.9	Fragment
					84.36	114.8	53.5	Fiber
WP4_KOR_V1_F_21	2.35	0	0.00	/				
WP4_KOR_V1_F_22	1.70	1	0.59	PS	75.79	80.2	19.1	Fiber
WP4_KOR_V1_F_23	3.26	0	0.00	/				
WP4_KOR_V1_F_24	2.78	0	0.00	/				
WP4_KOR_V1_F_25	1.50	0	0.00	/				
WP4_KOR_V1_F_26	4.39	0	0.00	/				
WP4_KOR_V1_F_27	2.23	3	1.35	PS	73.88	92	58.4	Spheroid
					79.94	109.9	78.6	Spheroid
					90.41	279.5	86.4	Fiber
WP4_KOR_V1_F_28	1.57	0	0.00	/				
WP4_KOR_V1_F_29	1.10	0	0.00	/				
WP4_KOR_V1_F_30	2.41	1	0.41	PP	86.43	198.3	48.2	Fiber

Table S6. Counting, identification and and characterization MPs in **clams from Croatia** by microFTIR. # - mass of soft tissue + intravalvular liquid mass (g). Note: The samples F001-F009, F011, F012 were not analyzed as silicon flters were broken immediately before µFTIR analysis. Polymer abbreviations are provided in legend of Table S6.

**Sample name**	**Mass of the content of shell (g) #**	**No. of MPs/ individual**	**No. of MPs /g of shell content**	**Type of MP**	**Match (%)**	**Length (µm)**	**Width (µm)**	**Shape**
WP4_SCH_V1_F010	1.5	0	0.00	/				
WP4_SCH_V1_F013	2.59	0	0.00	/				
WP4_SCH_V1_F014	3.38	0	0.00	/				

WP4_SCH_V1_F015	4.43	0	0.00	/				
WP4_SCH_V1_F016	7.42	0	0.00	/				
WP4_SCH_V1_F017	2.6	3	1.15	PP	83.89	171.3	56.8	Fragment
				PS	72.25	139.5	51.3	Fragment
				PP	70.87	209.4	99.2	Fragment
WP4_SCH_V1_F018	3.75	0	0.00	/				
WP4_SCH_V1_F019	2.66	0	0.00	/				
WP4_SCH_V1_F020	3.33	3	0.90	PP	71.61	209.4	99.2	Fragment
				PP	90.59	171.3	56.8	Fragment
				PS	72.7	139.3	51.3	Fragment
WP4_SCH_V1_F021	3.29	3	0.91	PP	78.12	85.2	55.6	Fragment
				PET	73.06	150.4	70.6	Fragment
				PU	77.72	39.2	16.3	Fragment
WP4_SCH_V1_F022	3.29	2	0.61	PP	92.54	152.8	75	Fragment
				PET	73.97	90.4	37.5	Fiber
WP4_SCH_V1_F023	1.7	1	0.59	PP	89.66	70.2	55.2	Fragment
WP4_SCH_V1_F024	6.24	1	0.16	PP	88.03	110.3	65	Fragment
WP4_SCH_V1_F025	4.68	0	0.00	/				
WP4_SCH_V1_F026	1.62	0	0.00	/				
WP4_SCH_V1_F027	5.68	0	0.00	/				
WP4_SCH_V1_F028	2.85	0	0.00	/				
WP4_SCH_V1_F029	5.84	0	0.00	/				
WP4_SCH_V1_F030	1.99	3	1.51	PS	81.98	56.7	21.7	Fragment
				PS	87.87	127.9	38.5	Fiber
				PS	87.87	74.6	36.4	Fragment
WP4_SCH_V1_F031	2.61	2	0.77	PE	71.48	54.1	46.7	Spheroid
				PS	75.91	29.5	18.8	Fragment
WP4_SCH_V1_F032	2.64	0	0.00	/				
WP4_SCH_V1_F033	1.3	1	0.77	PA	74.6	67.3	48	Fragment
WP4_SCH_V1_F034	6.48	3	0.46	PA	77.28	32.8	27.5	Spheroid
				PA	81.65	112.8	51	Fragment
				PS	70.09	37	28.1	Spheroid
WP4_SCH_V1_F035	4.02	0	0.00	/				
WP4_SCH_V1_F036	6.16	0	0.00	/				
WP4_SCH_V1_F037	7.21	0	0.00	/				
WP4_SCH_V1_F038	7.53	0	0.00	/				

WP4_SCH_V1_F039	5.76	0	0.00	/
WP4_SCH_V1_F040	3.03	0	0.00	/

Table S7. Counting, identification and and characterization MPs in **clams from Serbia** by microFTIR. # - mass of soft tissue + intravalvular liquid mass (g). Polymer abbreviations are provided in legend of Table S6.

Sample name	Mass of the content of shell (g) #	No. of MPs/individual	Number of MPs /g of shell content	Type of MP	Match (%)	Length (µm)	Width (µm)	Shape
WP4_SRB_1_FRESH_1	4.33	1.00	0.23	PP	93.32	236.5	75.6	Fragment
WP4_SRB_1_FRESH_2	3.57	0.00	0.00	/				
WP4_SRB_1_FRESH_3	4.85	1.00	0.21	PP	77.04	134.6	100.3	Fragment
WP4_SRB_1_FRESH_4	6.45	0.00	0.00	/				
WP4_SRB_1_FRESH_5	6.50	0.00	0.00	/				
WP4_SRB_1_FR_1	7.86	1.00	0.13	PP	87.64	257.6	197.2	Fragment
WP4_SRB_1_FR_2	7.08	0.00	0.00	/				
WP4_SRB_1_FR_3	7.41	2.00	0.27	PP	78.28	70.2	47.8	Fragment
				PS	72.91	161.9	65.1	Fragment
WP4_SRB_1_FR_4	6.41	2.00	0.31	PP	85.06	185.3	85.9	Fragment
				PE	92.29	110.1	104.2	Spheroid
WP4_SRB_1_FR_5	9.43	1.00	0.11	PE	91.97	162.5	66.7	Fragment
WP4_SRB_2_FRESH_1	8.40	1.00	0.12	PE	78.87	125.3	63.8	Fiber
WP4_SRB_2_FRESH_2	5.72	0.00	0.00	/				
WP4_SRB_2_FRESH_3	7.96	0.00	0.00	/				
WP4_SRB_2_FRESH_4	4.08	1.00	0.25	PS	87.87	230.6	46.6	Fiber
WP4_SRB_2_FRESH_5	8.95	1	0.11	Oxidized ethene homopolymer	72.1	60.2	31.9	Fragment

Table S8. Counting, identification and and characterization MPs in **mussels from South Korea** by microFTIR. # - mass of soft tissue + intravalvular liquid mass (g). Polymer abbreviations are provided in legend of Table S6.

Sample name	Mass of the content of shell (g) #	No. of MPs/ individual	No. of MPs /g of shell content	Type of MP	Match (%)	Length (µm)	Width (µm)	Shape
WP4_GK_Mc01_2Y_F_1	8.35	1	0.12	PS	74.01	50.1	28.1	Fiber
WP4_GK_Mc01_2Y_F_2	2.7	1	0.37	PE	91.34	275.7	143.1	Fragment
WP4_GK_Mc01_2Y_F_3	3.05	3	0.98	PE	81.74	177.2	112	Fragment
				PS	72.89	115.7	37.9	Fiber
				PS	82.12	97.3	26.3	Fiber
WP4_GK_Mc01_2Y_F_4	5.72	1	0.17	PE	91.62	233.2	157.2	Fragment
WP4_GK_Mc01_2Y_F_5	3.98	1	0.25	PS	87.87	313.4	18.9	Fiber
WP4_GK_Mc01_2Y_F_6	5.67	1	0.18	PS	87.87	60.7	23	Fragment
WP4_GK_Mc01_2Y_F_7	3.07	0.00	0.00	/				
WP4_GK_Mc01_2Y_F_8	2.72	1.00	0.37	Nylon	71.9	82	40.8	Fragment
WP4_GK_Mc01_2Y_F_9	4.14	0.00	0.00	/				
WP4_GK_Mc01_2Y_F_10	5.74	0.00	0.00	/				
WP4_GK_Mc01_2Y_F_11	4.08	1.00	0.25	PP	70.66	270.7	162.5	Fragment
WP4_GK_Mc01_2Y_F_12	2.65	1.00	0.38	PS	91.87	445.3	196.6	Fragment
WP4_GK_Mc01_2Y_F_13	2.50	1.00	0.40	PP	70.66	92.4	50	Fragment
WP4_GK_Mc01_2Y_F_14	3.31	0.00	0.00	/				
WP4_GK_Mc01_2Y_F_15	2.55	1	0.39	PP	93.7	200.5	131.3	Fragment
WP4_GK_Mc01_2Y_F_16	5.32	1	0.19	PP	70.66	270.7	124	Fragment
WP4_GK_Mc01_2Y_F_17	3.22	3	0.93	PS	85.99	45.1	29.8	Fragment
				PS	71.94	75.2	42.1	Fragment
				PS	79.72	50.1	35.1	Fragment
WP4_GK_Mc01_2Y_F_18	7.72	2	0.26	PET	77.45	190.5	42.3	Fiber
				PS	79.99	65.2	21.1	Fiber
WP4_GK_Mc01_2Y_F_19	7.63	1	0.13	PE	70.14	182.5	53.8	Fiber
WP4_GK_Mc01_2Y_F_20	3.26	0.00	0.00	/				
WP4_GK_Mc01_2Y_F_21	6.77	0.00	0.00	/				
WP4_GK_Mc01_2Y_F_22	2.90	0.00	0.00	/				

WP4_GK_Mc01_2Y_F_23	5.92	0.00	0.00	/				
WP4_GK_Mc01_2Y_F_24	3.03	1.00	0.33	PP	72.82	192.6	56.3	Fragment
WP4_GK_Mc01_2Y_F_25	3.04	1.00	0.33	PS	78.95	70.2	34.2	Fragment
WP4_GK_Mc01_2Y_F_26	5.01	0.00	0.00	/				
WP4_GK_Mc01_2Y_F_27	6.90	0.00	0.00	/				
WP4_GK_Mc01_2Y_F_28	3.29	0.00	0.00	/				
WP4_GK_Mc01_2Y_F_29	4.98	0.00	0.00	/				
WP4_GK_Mc01_2Y_F_30	3.27	1.00	0.31	PS	87.87	46.2	42.9	Spheroid

Table S9. Counting, identification and and characterization MPs in **mussels from Croatia** by microFTIR. # - mass of soft tissue + intravalvular liquid mass (g). Note: The samples F007, F014 and F017 were not analyzed as silicon flters were broken immediately before µFTIR analysis. Polymer abbreviations are provided in legend of Table S6.

**Sample name**	**Mass of the content of shell (g) #**	**No. of MPs/ individual**	**Number of MPs /g of shell content**	**Type of MP**	**Match (%)**	**Length (µm)**	**Width (µm)**	**Shape**
WP4_SCH_M1_F_001	3.04	1.00	0.33	CP	70.19	155.7	31.3	Fiber
WP4_SCH_M1_F_002	16.90	0.00	0.00	/				
WP4_SCH_M1_F_003	13.73	2	0.15	PP	85.14	120.3	28.4	Fragment
				PS	75.87	40.4	6.8	Fragment
WP4_SCH_M1_F_004	1.86	0	0.00	/				
WP4_SCH_M1_F_005	9.54	0	0.00	/				
WP4_SCH_M1_F_006	2.13	0	0.00	/				
WP4_SCH_M1_F_008	8.14	0	0.00	/				
WP4_SCH_M1_F_009	2.59	0	0.00	/				
WP4_SCH_M1_F_010	7.73	2	0.26	CP	71.18	233	10.3	Fiber
				PAN	72.07	80.2	37.1	Fragment
WP4_SCH_M1_F_011	5.31	1.00	0.19	CP	76.3	211.5	100.81	Fiber
WP4_SCH_M1_F_012	8.08	2	0.25	CP	79.98	75.2	21.2	Fiber
				CP	75.67	114.5	46.2	Fiber
WP4_SCH_M1_F_013	3.24	0	0.00	/				

WP4_SCH_M1_F_015	2.94	3	1.02	PP	91.19	134.6	128.2	Spheroid
				PP	85.95	102.5	97.6	Spheroid
				EVA	79.36	123	35.9	Fragment
WP4_SCH_M1_F_016	4.44	0	0.00	/				
WP4_SCH_M1_F_018	2.96	0	0.00	/				
WP4_SCH_M1_F_019	3	2	0.67	PET	79.84	466.2	34.3	Fragment
				CP	72.16	140.4	49.9	Fragment
WP4_SCH_M1_F_020	8.25	3	0.36	CP	70.53	240.6	76.4	Fragment
				PE	83.28	80.2	31.4	Fragment
				PS	74.17	50.1	11.6	Fiber
WP4_SCH_M1_F_021	2.81	3	1.07	PS	96.3	179.7	149	Fragment
				PS	92.02	97.4	96.7	Fragment
				PS	86.99	116.4	112.8	Fragment
WP4_SCH_M1_F_022	3.26	0	0.00	/				
WP4_SCH_M1_F_023	6.27	0	0.00	/				
WP4_SCH_M1_F_024	4.97	0	0.00	/				
WP4_SCH_M1_F_025	1.48	1.00	0.68	PS	91.65	273.1	0.47	Fragment
WP4_SCH_M1_F_026	4.28	0.00	0.00					
WP4_SCH_M1_F_027	3.34	1.00	0.30	CP	80.23	143.6	1.39	Fiber
WP4_SCH_M1_F_028	9.89	0.00	0.00	/				
WP4_SCH_M1_F_029	6.22	0.00	0.00	/				
WP4_SCH_M1_F_030	3.91	1.00	0.26	PE	89.74	102.5	51.3	Fragment

Table S10. Counting, identification and and characterization MPs in **mussels from Belgium** by microFTIR. # - mass of soft tissue + intravalvular liquid mass (g). Polymer abbreviations are provided in legend of Table S6.

**Sample name**	**Mass of the content of shell (g) #**	**No. of MPs/ individual for all samples**	**No. of MPs /g of shell content**	**Type of MP**	**Match (%)**	**Length (µm)**	**Width (µm)**	**Shape**
WP4_SCI_MO1_1_001	2.21	1	0.45	PS	72.68	87.4	67.7	Fragment

WP4_SCI_MO1_1_002	2.75	2	0.73	PVC	72.61	547.3	232.8	Fragment
				PP	84	608	124.9	Fragment
WP4_SCI_Me01_V1_F_3	3.34	1	0.30	PS	79.41	59.5	25.6	Fragment
WP4_SCI_Me01_V1_F_4	2.74	0	0.00	/				
WP4_SCI_Me01_V1_F_5	4.56	0	0.00	/				
WP4_SCI_Me01_V1_F_6	1.29	0	0.00	/				
WP4_SCI_Me01_V1_F_7	3.58	1	0.28	PET	87.37	344.7	118.3	Fragment
WP4_SCI_Me01_V1_F_8	2.94	1	0.34	PA	70.3	150.4	127	Spheroid
WP4_SCI_Me01_V1_F_9	3.1	0	0.00	/				
WP4_SCI_Me01_V1_F_10	5.34	0	0.00	/				
WP4_SCI_Me01_V1_F_11	4.74	0	0.00	/				
WP4_SCI_Me01_V1_F_12	3.44	0	0.00	/				
WP4_SCI_Me01_V1_F_13	4.23	0	0.00	/				
WP4_SCI_Me01_V1_F_14	3.83	1	0.26	Ny	77.48	50.1	22.3	Fragment
WP4_SCI_Me01_V1_F_15	4.08	1	0.25	PS	73.76	35.1	21	Fragment
WP4_SCI_Me01_V1_F_16	2.77	0	0.00	/				
WP4_SCI_Me01_V1_F_17	3.31	0	0.00	/				
WP4_SCI_Me01_V1_F_18	2.97	0	0.00	/				
WP4_SCI_Me01_V1_F_19	4.24	0	0.00	/				
WP4_SCI_Me01_V1_F_20	2.38	1	0.42	PE	81.63	156.5	65	Fiber
WP4_SCI_Me01_V1_F_21	2.77	1	0.36	PS	74.04	30.1	21.3	Spheroid
WP4_SCI_Me01_V1_F_22	3.32	0	0.00	/				
WP4_SCI_Me01_V1_F_23	2	0	0.00	/				
WP4_SCI_Me01_V1_F_24	3.01	0	0.00	/				
WP4_SCI_Me01_V1_F_25	2.35	0	0.00	/				
WP4_SCI_Me01_V1_F_26	1.82	0	0.00	/				

Table S11. Counting, identification and and characterization MPs in **Crustacea shellfish from South Korea** by microFTIR. Polymer abbreviations are provided in legend of Table S6.

Sample name	Mass of the content of shell (g)	No. of MPs/ individual	No. of MPs /g of shell content	Type of MP	Match (%)	Length (µm)	Width (µm)	Shape
WP4_GK_Lv01_2_F_1	13.07	0	0.00	/				
WP4_GK_Lv01_2_F_2	9.19	2	0.22	PET	82.83	358.3	198.5	Fragment
				PA	82.45	181.8	37.4	Fiber
WP4_GK_Lv01_2_F_3	9.08	0	0.00	/				
WP4_GK_Lv01_2_F_4	8.11	2	0.25	PVC	81.64	123	49.2	Fragment
				PA	87.72	158.9	74.7	Fragment
WP4_GK_Lv01_2_F_5	11.13	1	0.09	PA	90.18	157	59	Fragment
WP4_GK_Lv01_2_F_6	9.11	0	0.00	/				
WP4_GK_Lv01_2_F_7	12.31	0	0.00	/				
WP4_GK_Lv01_2_F_8	8.99	1	0.11	PA	79.97	335.9	59.5	Fiber
WP4_GK_Lv01_2_F_9	9.96	1	0.10	PET	71.59	113.4	58.4	Fragment
WP4_GK_Lv01_2_F_10	8.65	1	0.12	PET	79.44	87.7	54.4	Fragment
WP4_GK_Lv01_2_F_11	7.04	1	0.14	Nylon	81.42	52.2	23.3	Fragment
WP4_GK_Lv01_2_F_12	9.42	0	0.00	/				

Table S12. Counting, identification and and characterization MPs in **Crustacea shellfish from Croatia** by µFTIR. Note: The samples F002, F004 and F007 were not analyzed as silicon flters were broken immediately before µFTIR analysis. Polymer abbreviations are provided in legend of Table S6.

Sample name	Mass of the content of shell (g)	Number of MPs/ individual	No. of MPs /g of shell content	Type of MP	Match (%)	Length (µm)	Width (µm)	Shape
WP4_SCH_N1_F_001	13.33	0	0.00	/				
WP4_SCH_N1_F_003	12.23	0	0.00	/				
WP4_SCH_N1_F_005	5.96	0	0.00	/				
WP4_SCH_N1_F_007	3.64	0	0.00	/				
WP4_SCH_N1_F_008	5.1	1	0.20	PP	77.6	116.6	54	Fragment
WP4_SCH_N1_F_009	5.7	0	0.00	/				

WP4_SCH_N1_F_010	5.83	1	0.17	PVC	76.29	119.6	106.2	Spheroid
WP4_SCH_N1_F_011	7.04	1	0.14	PA	76.94	75.2	50.2	Fragment
WP4_SCH_N1_F_012	9.42	2	0.21	PVC	74.58	71.8	36.8	Fragment
				CP	79.76	84.1	52.1	Fragment

Table S13. Counting, identification and and characterization MPs in **Crustacea shellfish from Belgium** by μFTIR. Polymer abbreviations are provided in legend of Table S6.

**Sample name**	**Mass of the content of shell (g)**	**Number of MPs/individual**	**Number of MPs /g of shell content**	**Type of MP**	**Match (%)**	**Length (μm)**	**Width (μm)**	**Shape**
WP4_SCI_Lv_01_1	11.51	1.00	0.09	PA	89.57	144.3	52.3	Fiber
WP4_SCI_Lv_01_2	11.57	1.00	0.09	PA	90.56	89.2	57.4	Fragment
WP4_SCI_Lv_01_3	10.42	2.00	0.19	PE	91.09	171.7	123.9	Spheroid
				PP	85.67	672.8	257.3	Fragment
WP4_SCI_Lv_01_4	10.89	1.00	0.09	PET	92.27	627.7	59.8	Fiber
WP4_SCI_Lv_01_5	11.56	0.00	0.00	/				
WP4_SCI_Lv_01_6	10.41	2.00	0.19	PVC	71.85	115.3	45.2	Fragment
				PA	85.08	101.5	74.7	Fragment
WP4_SCI_Lv_01_7	8.05	0.00	0.00	/				
WP4_SCI_Lv_01_8	10.22	0.00	0.00	/				
WP4_SCI_Lv_01_9	10.30	0.00	0.00	/				
WP4_SCI_Lv_01_10	9.43	0.00	0.00	/				
WP4_SCI_Lv_01_11	13.78	0.00	0.00	/				
WP4_SCI_Lv_01_12	8.53	0.00	0.00	/				

Table S14. Overview of counting of MPs in different species from different regions.

**Parameter**	**Clams S. Korea**	**Clams Croatia**	**Clams Serbia**	**Mussels S. Korea**	**Mussles Croatia**	**Mussels Belgium**	**Crustacea shellfish S. Korea**	**Crustacea shellfish Croatia**	**Crustacea shellfish Belgium**
No. of individuals analyzed	30.00	29.00	15.00	30.00	27.00	26.00	12.00	9.00	12.00
No. of individuals with MPs found	9.00	10.00	9.00	18.00	12.00	9.00	7.00	4.00	5.00

% individuals containing MPs (Frequency of ingestion)	30.00	34.48	60.00	60.00	44.44	34.62	58.33	44.44	41.67
Total MPs found	15.00	22.00	11.00	23.00	22.00	10.00	9.00	5.00	7.00
Total mass of the content of shell analyzed (g)	76.89	115.58	99.00	132.49	150.31	83.11	116.06	68.25	126.67
No. of MPs /g of shell content (total no. of MPs found/total mass of content of shell analyzed)	0.20	0.19	0.11	0.17	0.15	0.12	0.08	0.07	0.06
No. of MPs /individual (total MPs found/total no. of individuals analyzed)	0.50	0.76	0.73	0.77	0.81	0.38	0.75	0.56	0.58

Table S15. Overview of mean values ± SD for counting and characterization of MPs in different species from different regions.

**Parameter (Mean ± SD)**	**Clams S. Korea**	**Clams Croatia**	**Clams Serbia**	**Mussels S. Korea**	**Mussels Croatia**	**Mussels Belgium**	**Crustacea shellfish S. Korea**	**Crustacea shellfish Croatia**	**Crustacea shellfish Belgium**
Mass of content of shell (g)	2.56 ± 0.78	3.99 ± 1.91	6.60 ± 1.81	4.42 ± 1.76	5.57 ± 3.73	3.20 ± 0.95	9.67 ± 1.72	7.58 ± 3.34	10.56 ± 1.52
No. of MP/g of shell content in all tested individuals	0.21 ± 0.39	0.27 ±0.43	0.12 ± 0.11	0.21 ± 0.25	0.20 ± 0.31	0.13 ± 0.20	0.09 ± 0.09	0.08 ± 0.10	0.05 ± 0.08
No. of MPs/g of shell content for subgroup containing MPs	0.71 ±0.38	0.78 ± 0.37	0.18 ± 0.08	0.35± 0.24	0.46 ± 0.32	0.38 ± 0.15	0.15 ± 0.06	0.18 ± 0.03	0.13 ± 0.05
No. of MP/individual in all tested individuals	0.50 ± 0.97	0.76 ± 1.18	0.73 ± 0.70	0.77± 0.82	0.81 ± 1.08	0.38 ± 0.57	0.75 ± 0.75	0.56 ± 0.73	0.58 ± 0.79
No. of MPs/ individual for subgroup containing MPs	1.67 ± 1.12	2.20 ± 0.92	1.22 ± 0.44	1.28 ± 0.67	1.83 ± 0.83	1.11 ± 0.33	1.29 ± 0.49	1.25 ± 0.50	1.40 ± 0.55
Match %	86.23 ± 7.64	79.26 ± 7.38	83.40 ± 7.85	80.16 ± 8.14	80.90 ± 7.99	77.33 ± 5.63	81.92 ± 5.24	77.03 ± 1.89	86.58 ± 7.04

Length (um)	166.27 ± 82.37	105.97 ± 56.42	157.71 ± 65.82	156.63 ± 107.30	149.14 ± 93.58	206.91 ± 216.75	174.24 ± 105.66	93.46 ± 22.96	274.64 ± 258.37
Width (um)	66.13 ± 21.42	50.30 ± 22.52	80.46 ± 44.62	69.97 ± 54.76	52.69 ± 43.52	82.59 ± 68.77	68.27 ± 50.98	59.86 ± 26.77	95.80 ± 75.87

Table S16. Overview of ranges for counting and characterization of MPs in different species from different regions.

**Parameter range (Min - Max)**	**Clams S. Korea**	**Clams Croatia**	**Clams Serbia**	**Mussels S.Korea**	**Mussles Croatia**	**Mussels Belgium**	**Crustacea shellfish S. Korea**	**Crustacea shellfish Croatia**	**Crustacea shellfish Belgium**
Mass of content of shell (g)	1.10 - 4.39	1.30 - 7.53	3.57 - 9.43	2.50 - 8.35	1.48 - 16.90	1.29 - 5.34	7.04 - 13.07	3.64 - 3.33	8.05 - 13.78
No. of MP/g of shell content in all tested individuals	0.00 - 1.35	0.00 - 1.51	0.00 - 0.31	0.00 - 0.98	0.00 - 1.07	0.00 - 0.73	0.00 - 0.25	0.00 - 0.21	0.00 - 0.19
No. number of MPs/g of shell content for subgroup containing MPs	0.33 - 1.35	0.16 - 1.51	0.11 - 0.31	0.12 - 0.98	0.15 - 1.07	0.25 - 0.73	0.09 - 0.25	0.14 - 0.21	0.09 - 0.19
No. of MP/individual in all tested individuals	0.00 - 4.00	0.00 - 3.00	0.00 - 2.00	0.00 - 3.00	0.00 - 3.00	0.00 - 2.00	0.00 - 2.00	0.00 - 2.00	0.00 - 2.00
No. of MPs/ individual for subgroup containing MPs	1.00 - 4.00	1.00 - 3.00	1.00 - 2.00	1.00 - 3.00	1.00 - 3.00	1.00 - 2.00	1.00 - 2.00	1.00 - 2.00	1.00 - 2.00
Range of match %	73.88 - 98.09	70.09 - 92.54	72.10 - 93.32	70.14 - 93.70	70.19 - 96.30	70.30 - 87.37	71.59 - 90.18	74.58 - 79.76	71.85 - 92.27
Range of length (um)	80.20 - 322.20	29.50 - 209.40	60.20 - 257.60	45.10 - 445.30	0.47 - 466.20	30.10 - 608.00	52.20 - 358.30	71.80 - 119.60	89.20 - 672.80
Range of width (um)	19.10 - 76.89	16.30 - 115.58	31.90 - 197.20	18.90 - 196.60	6.80 - 273.10	21.00 - 232.80	23.30 - 198.50	36.80 - 106.20	45.20 - 257.30

Table S17. Overview of shapes and types of MPs in different species from different regions.

	**Clams S. Korea**	**Clams Croatia**	**Clams Serbia**	**Mussels**	**Mussles**	**Mussels**	**Crustacea**	**Crustacea**	**Crustacea**

				**S.Kore a**	**Croati a**	**Belgiu m**	**shellfish S. Korea**	**shellfish Croatia**	**shellfish Belgium**
No. of fragments	6.00	17.00	8.00	15.00	13.00	7.00	7.00	4.00	4.00
No. of fibers	5.00	2.00	2.00	7.00	7.00	1.00	2.00	0.00	2.00
No. of sheroids	4.00	3.00	1.00	1.00	2.00	2.00	0.00	1.00	1.00
No. of PS MPs	6.00	7.00	2.00	12.00	6.00	4.00	0.00	0.00	0.00
No. of PP MPs	7.00	8.00	5.00	5.00	3.00	1.00	0.00	1.00	1.00
No. of PA MPs	0.00	3.00	0.00	0.00	0.00	1.00	4.00	1.00	3.00
No. of CP MPs	1.00	0.00	0.00	0.00	8.00	0.00	0.00	1.00	0.00
No. of PET MPs	0.00	2.00	0.00	1.00	1.00	1.00	3.00	0.00	1.00
No. of PE MPs	1.00	1.00	3.00	4.00	2.00	1.00	0.00	0.00	1.00
No. of PVC MPs	0.00	0.00	0.00	0.00	0.00	1.00	1.00	2.00	1.00
No. of Ny MPs	0.00	0.00	0.00	1.00	0.00	1.00	1.00	0.00	0.00
No. of PAN MPs	0.00	0.00	0.00	0.00	1.00	0.00	0.00	0.00	0.00
No. of EVA MPs	0.00	0.00	0.00	0.00	1.00	0.00	0.00	0.00	0.00
No. of PU MPs	0.00	1.00	0.00	0.00	0.00	0.00	0.00	0.00	0.00
Other types	0.00	0.00	1.00	0.00	0.00	0.00	0.00	0.00	0.00

Table S18. The significance of microplastic transfer interactions for MP abundance of studied species according to statistical analyses.

Statistical Analysis Test	Parameter	Factor	p-value
**Collection: all measurements**			
Kruskal–Wallis	Clams	*Croatia – Korea*	0.9882
Kruskal–Wallis	Mussels	*Croatia – Korea - Belgium*	0.1056
Kruskal–Wallis	Crustacea shellfish	*Croatia – Korea - Belgium*	0.2955
Kruskal–Wallis	Korea	*clams– mussels - Crustacea shellfish*	0.2009
Kruskal–Wallis	Croatia	*clams– mussels - Crustacea shellfish*	0.4641
Mann–Whitney	Belgium	*mussels - Crustacea shellfish*	0.7605
	All	*clams– mussels - Crustacea shellfish*	0.217
**Subcollection: contaminated samples**			
Kruskal-Walllis		*Clams-mussels- Crustacea shellfish*	0.00000148
Mann-Whitney		*Clams-mussels*	0.01681
Mann-Whitney		*Clams- Crustacea shellfish*	0.000017
Mann-Whitney		*Mussels - Crustacea shellfish*	0.001468

References

1. de Guzman, M.K.; Andjelković, M.; Jovanović, V.; Jung, J.; Kim, J.; Dailey, L.A.; Rajković, A.; De Meulenaer, B.; Ćirković Veličković, T. Comparative Profiling and Exposure Assessment of Microplastics in Differently Sized Manila Clams from South Korea by MFTIR and Nile Red Staining. *Mar Pollut Bull* 2022, *181*, 113846, doi:10.1016/J.MARPOLBUL.2022.113846.
2. Dehaut, A.; Cassone, A.L.; Frère, L.; Hermabessiere, L.; Himber, C.; Rinnert, E.; Rivière, G.; Lambert, C.; Soudant, P.; Huvet, A.; et al. Microplastics in Seafood: Benchmark Protocol for Their Extraction and Characterization. *Environmental Pollution* 2016, *215*, 223–233, doi:10.1016/J.ENVPOL.2016.05.018.
